# A novel gene-diet pair modulates *C*. *elegans* aging

**DOI:** 10.1371/journal.pgen.1007608

**Published:** 2018-08-20

**Authors:** Sonia Verma, Urmila Jagtap, Anita Goyala, Arnab Mukhopadhyay

**Affiliations:** Molecular Aging Laboratory, National Institute of Immunology, Aruna Asaf Ali Marg, New Delhi, India; Cornell University, UNITED STATES

## Abstract

Diet profoundly affects metabolism and incidences of age-related diseases. Animals adapt their physiology to different food-types, modulating complex life-history traits like aging. The molecular mechanisms linking adaptive capacity to diet with aging are less known. We identify FLR-4 kinase as a novel modulator of aging in *C*. *elegans*, depending on bacterial diet. FLR-4 functions to prevent differential activation of the p38MAPK pathway in response to diverse food-types, thereby maintaining normal life span. In a kinase-dead *flr-4* mutant, *E*. *coli* HT115 (K12 strain), but not the standard diet OP50 (B strain), is able to activate p38MAPK, elevate expression of cytoprotective genes through the nuclear hormone receptor NHR-8 and enhance life span. Interestingly, *flr-4* and dietary restriction utilize similar pathways for longevity assurance, suggesting cross-talks between cellular modules that respond to diet quality and quantity. Together, our study discovers a new *C*. *elegans* gene-diet pair that controls the plasticity of aging.

## Author summary

For animals living in the wild, being able to utilize a wide range of diet is evolutionarily advantageous as they can survive even when their optimal diet is depleted. Since diet is known to influence the rate of aging, animals seem to have evolved intricate mechanisms to maintain homeostasis and normal life span, but the molecular mechanisms are less understood. Using a small nematode, *C*. *elegans* as a model, we show that the adaptive capacity to different diet is maintained by a kinase gene. When this gene is mutated, worms start living longer on one strain of bacterial diet but not on the other. We identify the molecular cascade required for this food-type-dependent longevity. We show that this cascade of events significantly overlaps with the pathway that determine food quantity-dependent life span enhancement. Our study thus elucidates a part of the molecular monitoring system that regulates longevity dependent on the available quality and quantity of diet.

## Introduction

Animals dwell in a complex ecosystem where they interact with a host of other organisms; some of them may alter their life history traits. For example, the nematode *Caenorhabditis elegans* is found in decaying moist vegetation that is co-inhabited by different types of bacteria that they feed on. So, the worms are often exposed to various pathogenic bacteria that they either avoid or use a conserved innate immunity pathway to counter. The worms are also presented with a range of bacteria of different nutritional values that they choose between [1–3]. They encounter *E*. *coli*¸ *Bacillus* and *Comamonas* etc. that are known to modulate development, reproduction, fat storage and life span [2, 4–7]. In the laboratory, worms are mostly maintained on *E*. *coli* OP50 but are often exposed to the RNAseIII-deficient HT115 during RNAi experiments. The two strains differ considerably, particularly in terms of carbohydrate content, with the OP50 strain considered as a low quality diet that induces less satiety and promotes higher fat storage [3, 6–9]. Since the rate of aging is greatly influenced by dietary composition, worms seem to have evolved intricate adaptive strategies to maintain normal aging [10, 11]. As a result, although the two diets differentially affect metabolism, the worms are able to ensure relatively normal life span when fed either bacteria [7]. How *C*. *elegans* sense different diet to alter metabolism and life history traits, including complex traits like aging, is an emerging area of research. These studies are being facilitated by the discovery of gene-diet pairs where the function of a gene becomes discernible only on a particular diet [12].

Previous work has shown that sensory neurons may process signals that differentiate between different food-types and regulate life span in flies and worms [10, 13, 14]. In *C*. *elegans*, the neuromedin U receptor-like gene, *nmur-1* mutant as well as *osm-3* (kinesin motor protein required for cilia formation) mutant has extended life span on OP50 but not on HT115 [10, 14]. These life spans were found to be dependent on the FOXO transcription factor, DAF-16 [14]. On the other hand, the proline metabolism gene *alh-6* works in the muscle to preserve mitochondrial structure and functional homeostasis in response to OP50 [11]. This requires a functional NMUR-1 receptor signalling [11]. Interestingly and intuitively, it would appear that the intestine may also contribute to this phenomenon as it gets to sample different food that the worms ingest. However, the role of intestine in food-type-dependent life span regulation is not as well-characterized.

Here we show that a serine-threonine kinase gene, *flr-4* regulates food-type-dependent life span by functioning both in the neurons and the intestine. We find that when *flr-4* is knocked down by RNAi or its function disrupted by a P223S missense mutation in its kinase domain, life span is dramatically increased. The life span of the kinase-dead mutant *flr-4(n2259)* is increased only when the mutant is fed HT115 and not OP50. We show that knocking down *flr-4* leads to increased cytoprotective xenobiotic detoxification pathway (XDP) gene expression, through the nuclear hormone receptor NHR-8, that plays a causal role in its increased life span. Interestingly, this elevated gene expression as well as the increased life span is dependent on the conserved p38 MAPK signalling. In *flr-4(n2259)*, OP50 is unable to strongly activate the p38 MAPK while HT115 leads to increased phosphorylation of the MAPK. Consequently, in the mutant, HT115 is able to increase the levels of the XDP genes while OP50 does not. Finally, we demonstrate that FLR-4 uses a pathway similar to DR to ensure longevity, dependent on FOXA/PHA-4 but independent of FOXO/DAF-16. Together, our study establishes *flr-4* as a new longevity gene that controls adaptive capacity of *C*. *elegans* towards bacterial diet by preventing differential activation of XDP genes through p38MAPK pathway, dependent on food-type.

## Results

### Knocking down *flr-4* increases life span in a food-type dependent manner

**Fig 1:**
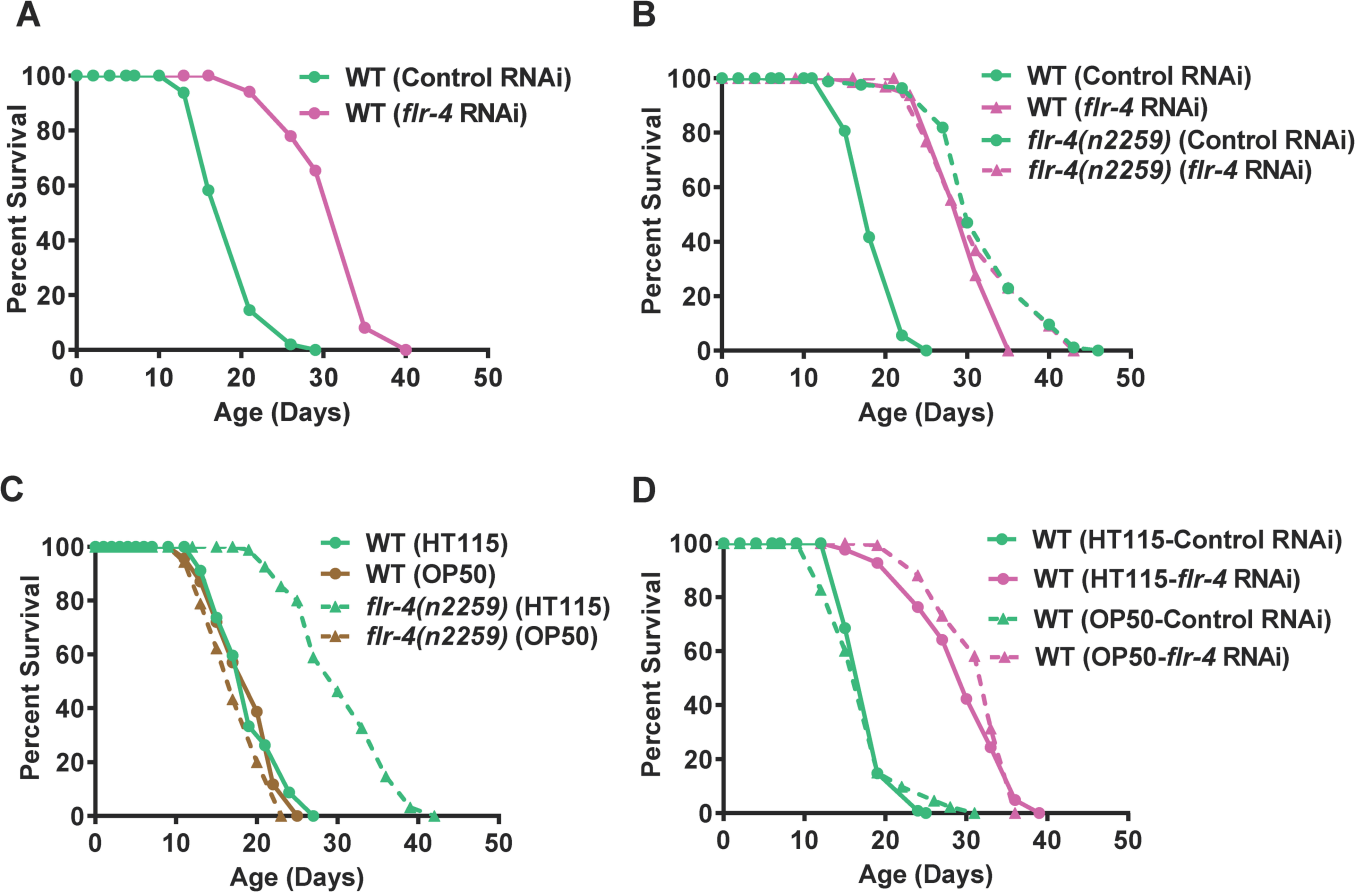
Knocking down *flr-4* increases life span in a food-type-dependent manner. (A) Knocking down *flr-4* by RNAi increases life span. (B) The *flr-4(n2259)* allele has increased life span that is not further extended when *flr-4* is knocked down using RNAi. (C) The *flr-4(n2259)* allele has increased life span only when the mutant worms are maintained on *E*. *coli* HT115 but not on *E*. *coli* OP50. (D) Knocking down *flr-4* using an OP50-based RNAi system increased life span comparable to HT115-based RNAi. Life spans were performed at 20°C.

*Flr-4* was originally identified in a screen for genes that regulate fluoride resistance and the mutants exhibit temperature-sensitive defecation defects [15]. However, at 20 ^o^C they do not have defects in defecation [15]. We initially became interested in the *flr-4* gene as it has 26% homology and 40% identity to *drl-1* [16], a gene we have recently characterized to be involved in Dietary Restriction (DR). Knocking down *flr-4* using a cDNA RNAi construct increased life span dramatically (average life span increase 40–60%, Fig 1A, S1 and S2 Tables). Similar life span extension was observed in absence of FUDR, a DNA synthesis inhibitor used to arrest confounding effects of progeny population during life span analysis (S1A Fig). The increased life span was also associated with better health as evident from lower lipofuscin pigment accumulation (S2A Fig), lesser muscular atrophy with age (S2B Fig) and consequently, better motility (S2C Fig). These worms were smaller in size (S2D Fig) but did not have any major defects in developmental rates (S3 Fig). However, the increase in life span was not associated with enhanced tolerance towards heat stress (S4B Fig); UV stress tolerance was only increased 13–15% compared to 40–60% increase in life span (S4A Fig). The increased life span of *flr-4* knock down was also not dependent on the heat shock transcription factor *hsf-1* (S4C and S4D Fig). Thus, *flr-4* seems to decouple longevity from stress resistance and is a novel longevity modulator.

Next, we asked whether an *flr-4* mutant has attributes similar to the *flr-4* RNAi. We used the *flr-4(n2259)* allele that has a P223S missense mutation in the activation loop of the protein kinase domain and shows increased fluoride resistance [15]. This allele has similar developmental rates as wild-type (S5 Fig) and shows no dauer arrest [15]. We found that *flr-4(n2259)* increased life span that was not further increased when grown on *flr-4* RNAi (Fig 1B, S1 and S2 Tables). The life span of the mutant was also not affected by the presence of FUDR (S1B Fig). This suggests that *flr-4(n2259)* behaves as a null allele to regulate life span.

*C*. *elegans* is known to respond differentially to bacterial diet to modulate life history traits, including life span [5–7, 14, 17]. Interestingly, we found that the life span extension in *flr-4(n2259)* is dependent on the food-type. When the mutant was grown on the *E*. *coli* HT115 (a K12 strain), life span was dramatically extended (Fig 1C, S1 and S2 Tables). In contrast, when grown on *E*. *coli* OP50 (a B strain), no extension of life span was observed (Fig 1C). We checked for differences in pumping under these conditions and found no change (S2E Fig). Also, ingestion of RFP-labelled beads was similar in WT and *flr-4(n2259)* (S2F Fig), suggesting that the differences in life span are not due to altered feeding behaviour. Furthermore, the developmental rate was similar in both the strains when grown on the two *E*. *coli* strains (S5 Fig). Remarkably, knocking down *flr-4* using an OP50-based RNAi system [18] increased life span similar to that of the HT115-based system (Fig 1D), suggesting that the food-type-dependent life span extension may be attributed to the kinase function of FLR-4. This conclusion is further supported by the observation that rescuing *flr-4(n2259)* with a kinase-proficient wild-type transgene suppresses the increased life span on HT115 (S16 Fig). Together, FLR-4 kinase suppresses pro-longevity cues in a food-type dependent manner.

### *Flr-4* functions in the intestine and neurons, during larval development to regulate adult life span

**Fig 2:**
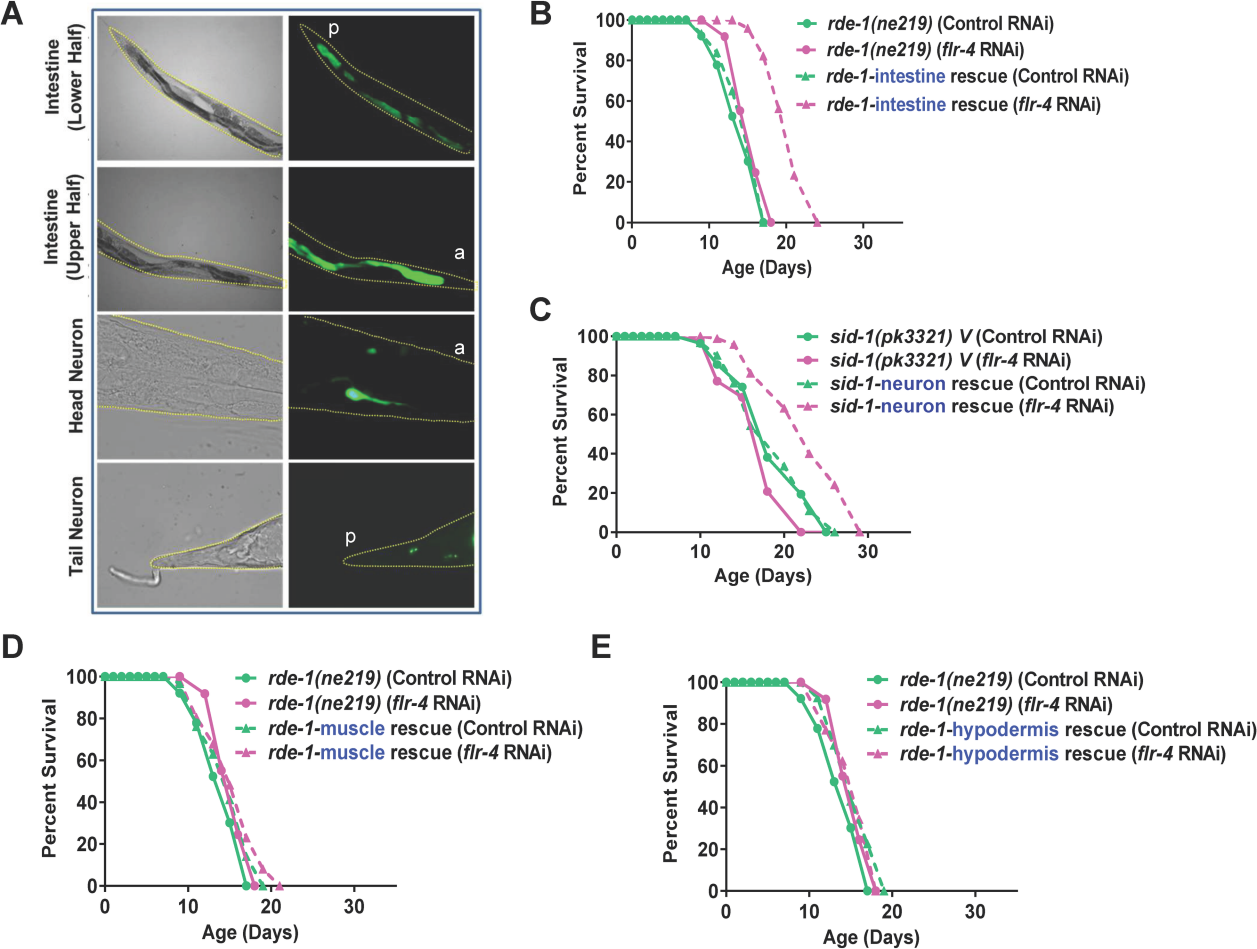
FLR-4 functions in the intestine and neurons to regulate life span. (A) The expression of *Pflr-4*::*gfp* is localized to the intestine and a few neurons. The anterior (a) or the posterior (p) ends of the worms are marked. Upper two panel 100X, lower two panels 630X magnification. L4 stage worms shown. (B-E) The *flr-4* RNAi had limited effect on the life span of RNAi-deficient *rde-1(ne219)*. Life span is extended only in *rde-1(ne213);kbIs7* where the *rde-1* gene is rescued in the intestine of *rde-1(ne219)* or in *sid-1(pk3321);uIs69* where *sid-1* is rescued in the neurons of *sid-1(pk3321)*. No extension was noticed in *rde-1(ne219);kzIs20* (muscle rescue) or *rde-1(ne219);kzIs9* (hypodermis rescue). Life spans were performed at 20 ^o^C.

Next, we asked where FLR-4 functions to regulate longevity. We constructed a *flr-4p*::*gfp* transgenic line and found that the *flr-4* promoter drove expression of *gfp* in the intestine and a few neurons (Fig 2A). We used a tissue-specific RNAi system to determine the tissue where *flr-4* functions. We found that *flr-4* knockdown specifically in the intestine or the neurons was sufficient for life span extension; no extension in life span was observed when the gene is solely knocked down in muscle or hypodermis (Fig 2B–2E, S1 and S2 Tables). Together, *flr-4* functions in the intestine and neurons to negatively regulate life span of the worms.

In *C*. *elegans*, longevity genes need to be knocked down at temporally distinct points in development to increase life span. For example, the mitochondrial electron transport gene *cco-1* or the MEKK-3-like kinase *drl-1* needs to be knocked down early in development to increase life span while insulin-like signalling pathway functions in adulthood to exhibit the beneficial longevity effects [16, 19, 20]. We initiated *flr-4* RNAi at different stages of development of the worms and found that knocking down at L1 or L2 produced maximum life span extension; knocking down at L3 or later had diminished or no effect (S6A–S6E Fig). Together, *flr-4* functions in the intestine and neurons, during larval development to regulate adult life span.

### *Flr-4* requires p38 MAPK pathway for longevity

**Fig 3:**
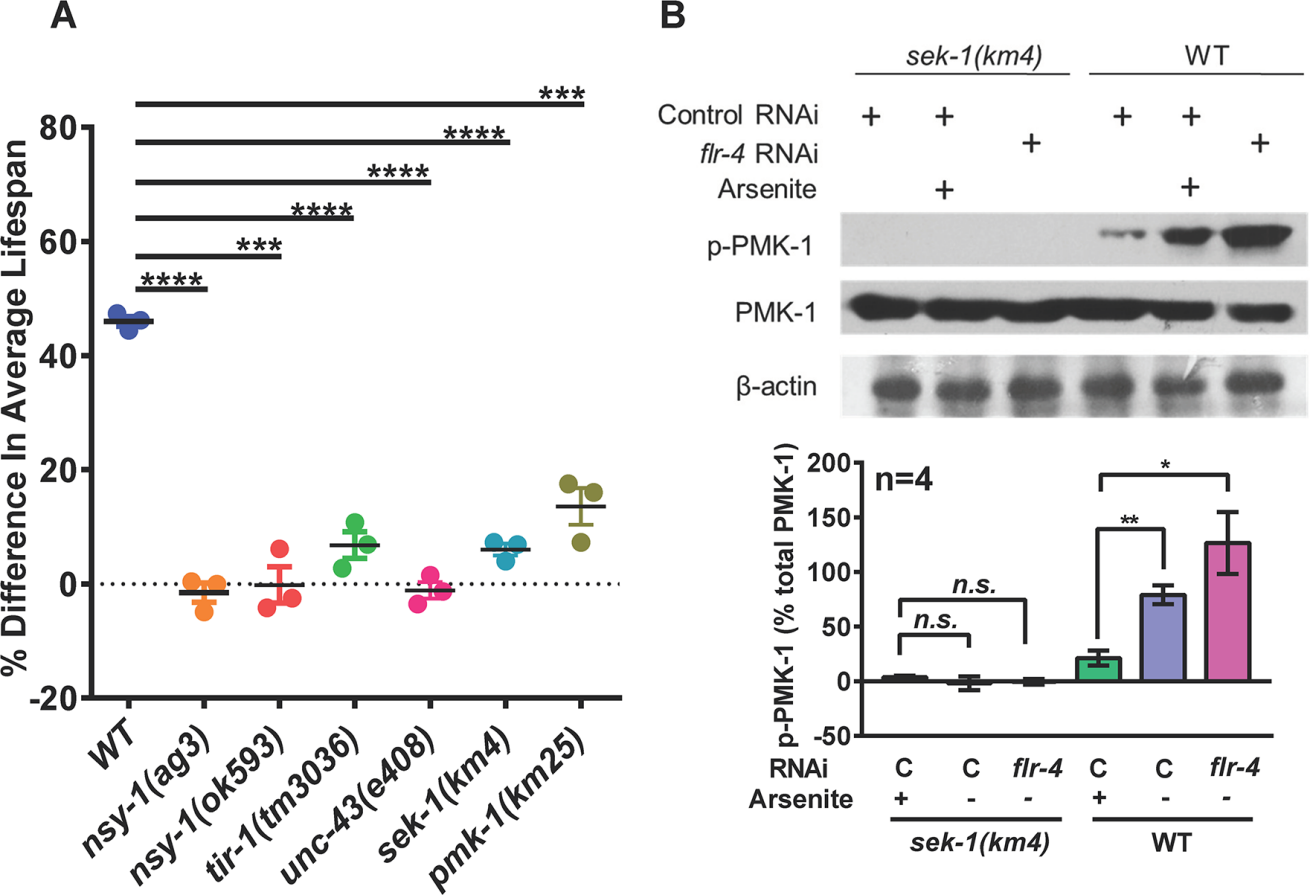
FLR-4 requires p38 MAPK pathway components to regulate life span. (A) The *flr-4* RNAi increased life span only in WT, but not to the same extent in *tir-1(tm3036)*, *unc-43(e408)*, *nsy-1(ag3)*, *nsy-1(ok593)*, *sek-1(km4)* or *pmk-1(km25)* mutants. The percent change in average life span on *flr-4* RNAi compared to control RNAi is plotted on the Y-axis. Error bar indicates SEM. ****P≤0.0001, ***P≤0.001, Student’s *t* test. All life spans were performed at 20 ^o^C. (B) Western blot analysis of young-adult WT or *sek-1(km4)* grown on control or *flr-4* RNAi using anti-phospho-PMK-1, anti-total PMK-1 or anti-β-actin antibodies. Quantification of the blot is shown below. The intensity of pPMK-1 and PMK-1 bands were normalized to beta-actin bands. Percent intensity of pPMK-1 with respect to total PMK is plotted. Average of 4 experiments shown. Error bar indicates SEM. **P≤0.01, *P≤0.05, n.s. not significant, Student’s *t* test. The activation of p38 MAPK pathway with 20 mM Arsenite was used as a control. Blots from 4 biological replicates available in S14 Fig.

Since FLR-4 is required to suppress the effect of food-type on longevity, we asked what signalling cascade may be mediating this effect. The p38 MAPK pathway is a central signalling mediator required for mounting the innate immune response when worms are challenged with pathogens [21–25]. The worm p38 homolog PMK-1 is activated by its upstream MAPKKK NSY-1 and MAPKK SEK-1 [26, 27]. The TIR domain adaptor protein TIR-1, an ortholog of human SARM [28, 29] and UNC-43, a Ca^2+^/calmodulin-dependent protein kinase II (CaMKII) [30] lie further upstream of these serine-threonine kinases. While TIR-1 works upstream of the p38 MAPK pathway to regulate innate immunity genes [28, 29], both TIR-1 and UNC-43 functions in the neurons to control neuronal cell fate and asymmetric patterns of odorant receptor expression [30, 31]. We grew wild-type, *pmk-1(km25)*, *sek-1(km4)*, *nsy-1(ag3)*, *nsy-1(ok593)*, *tir-1(tm3036)* and *unc-43(e403)* on control or *flr-4* RNAi and performed life span analysis. We found that increased life span on *flr-4* RNAi is suppressed when any of the kinases in the p38 MAPK pathway or *tir-1* or *unc-43* is mutated (Fig 3A, S1 and S2 Tables). Interestingly, *pmk-3* is not required for the life span extension, showing specificity of the process (S1 and S2 Tables). Similar suppression of life span was observed when *flr-4(n2259)* was grown on *sek-1* RNAi (S7A Fig). We also created a *flr-4(n2259)*:*sek-1(km4)* double mutant and found that the life span was suppressed (S7B Fig). Further, in order to determine biochemically whether knocking down *flr-4* activates the p38 MAPK, we performed western blot analysis using a phospo-PMK-1-specific antibody (Figs 3B and S14). We found that in WT worms when *flr-4* is knocked down, PMK-1 phosphorylation increases in a *sek-1*-dependent manner. Further, TIR-1 or UNC-43 seems to be working in the same linear pathway as *flr-4*; in the *tir-1(3036)* and *unc-43*(*e408)*, *flr-4* knockdown failed to increase phosphorylation of PMK-1 (S7C Fig). Together, the FLR-4 longevity signals are mediated by the p38 MAPK pathway.

### *Flr-4* knockdown activates XDP genes in a p38 MAPK-dependent manner

**Fig 4:**
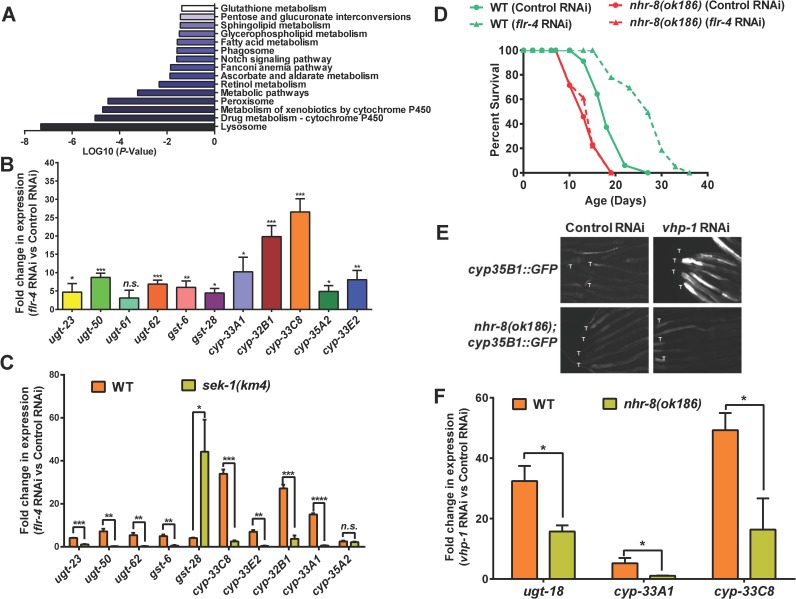
Knocking down *flr-4* transcriptionally activates the XDP genes in a p38 MAPK-dependent manner. (A) Enrichment of genes involved in xenobiotic metabolism among those that are upregulated when *flr-4* is knocked down using RNAi in WT, in comparison to control RNAi. Gene expression profiling was performed using RNA-seq and GO analysis performed using DAVID. Day 1 adult worms were used for RNA-seq. (B) Quantitative RT-PCR validation of expression of selected xenobiotic detoxification pathway (XDP) genes that were found to be upregulated using RNA-seq. (C) The expression of XDP genes is upregulated in WT in a *sek-1*-depdendent manner when *flr-4* is knocked down. (D) In the *nhr-8(ok168)* mutant, life span is not extended when *flr-4* is knocked down. (E) Knocking down *vhp-1* using RNAi induced expression of GFP in *cyp-35B1p*::*gfp* but not in the *nhr-8(ok186);cyp-35B1p*::*gfp* transgenic worms. Images are of worms 48 hours post L4. Tails are marked by ‘T’. Images captured at 100x magnification. (F) The expression of *ugt-18*, *cyp-33A1* and *cyp-33C8* are not upregulated in *nhr-8(ok186)* to the same extent as in WT, when *vhp-1* is knocked down using RNAi. Day 1 adult worms were used for RNA-seq and QRT-PCR. Error bar indicates SEM. ****P≤0.0001, ***P≤0.001, **P≤0.01, *P≤0.05, n.s. not significant, Student’s *t* test.

In order to understand how *flr-4* knockdown increases life span, we performed transcriptomic analysis of wild-type worms grown on control or *flr-4* RNAi. We found that 1957 genes were upregulated (> 2 folds, P ≤ 0.05) while 538 genes were down-regulated. We determined the biological functions of these genes using DAVID [32] and show that they are significantly enriched for genes involved in the Xenobiotic Detoxification Pathway (XDP) (Figs 4A and S8A). All these cytoprotective XDP genes are expressed in the intestine of the worms (www.wormbase.org). Using quantitative reverse transcriptase PCR (qRT-PCR), we verified 13 genes that were upregulated in our RNA-seq experiment (Figs 4B and S8B). We also used a transgenic worm expressing *gfp* driven by the *cyp-35B1* promoter and show that the expression of GFP is enhanced in the hind-gut region, when the worms were grown on *flr-4* RNAi (S8C Fig).

Next, in order to determine whether p38 MAPK pathway has any role in the regulation of these cytoprotective genes, we performed qRT-PCR analysis of these genes after knocking down *flr-4* in *sek-1(km4)*. Interestingly, majority of the XDP genes that were upregulated in the wild-type failed to do so in *sek-1(km4)* (Figs 4C and S8D). Since the levels of some of the genes do not fall back to basal level, it is possible that other signalling pathways or transcriptional regulators are also involved. These experiments suggested that p38 MAPK pathway regulates XDP genes downstream of *flr-4*.

### Suppressing XDP genes prevent *flr-4*-mediated life span extension

In order to determine whether XDP genes are indeed required for life span extension brought about by *flr-4* knockdown, we used a mutant of *nhr-8*, a transcription factor required for XDP gene expression [16, 33]. First, we knocked down *flr-4* by RNAi in *nhr-8(ok186)* and found that it failed to increase life span to the same extent as in wild-type (Fig 4D). We also knocked down *nhr-8* using RNAi in *flr-4(n2259)*, and found that the life span of the mutant is significantly suppressed (S8E Fig). Finally, we show that in *nhr-8(ok186)*, the XDP genes fail to upregulate to the same extent as in WT, when *flr-4* is knocked down (S8F Fig). Thus, increased expression of XDP genes is required for life span extension by *flr-4* knockdown.

Next, we asked whether NHR-8 functions downstream of p38 MAPK pathway to regulate XDP genes. For this, we decoupled p38 MAPK from *flr-4* and activated it by knocking down the phosphatase VHP-1 using RNAi [34, 35]. Knocking down *vhp-1* led to upregulation of *cyp-35B1*, as measured by increased GFP expression in the *cyp-35B1p*::*gfp* transgenic line. This enhancement was suppressed in the *cyp-35B1p*::*gfp;nhr-8(ok1853)* worms, showing that NHR-8 functions downstream of p38MAPK (Fig 4E). QRT-PCR analysis showed that three other XDP genes that are upregulated on *vhp-1* knockdown are dependent of NHR-8 (Fig 4F). Interestingly, knocking down *vhp-1* was not sufficient to increase life span of WT (S8G Fig), suggesting that additional downstream events may have to be coactivated in order to get life span benefits similar to *flr-4* knockdown.

### Diet-specific activation of p38 MAPK and XDP genes in *flr-4(n2259)*

**Fig 5:**
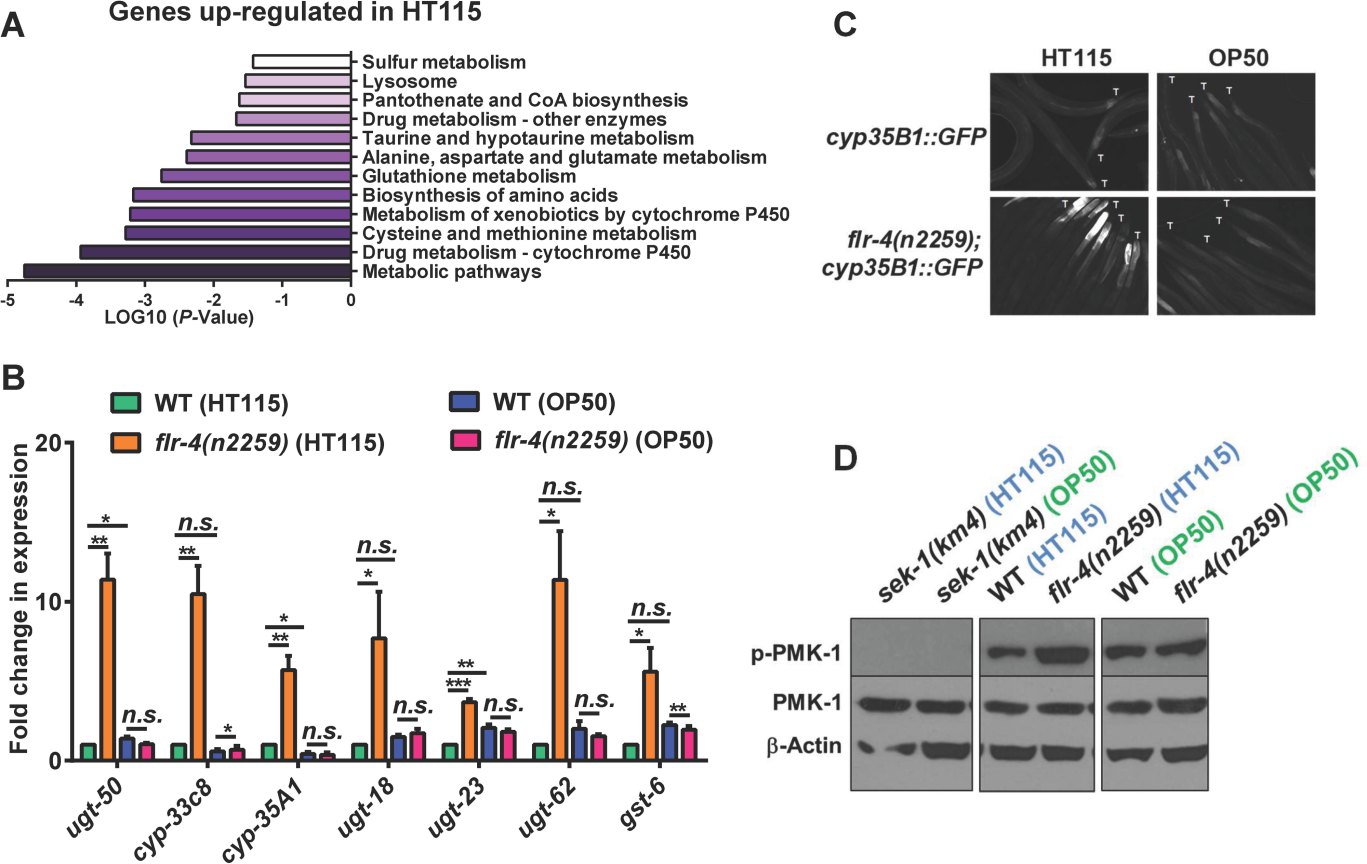
HT115 activates p38 MAPK pathway in *flr-4(n2259)*. (A) RNA-seq of *flr-4(n2259)* shows that genes involved in xenobiotic detoxification pathway (XDP) are upregulated only when worms were fed *E*. *coli* HT115. RNA from WT or *flr-4(n2259)* worms grown on HT115 or OP50 were used for sequencing. Genes upregulated on HT115 but not on OP50 were analyzed using DAVID. (B) Quantitative RT-PCR validation of RNA-seq data for selected XDP genes. These genes were upregulated only when *flr-4(n2259)* worms were fed HT115. Error bar indicates SEM. ****P≤0.0001, ***P≤0.001, **P≤0.01, *P≤0.05, n.s. not significant, Student’s *t* test. (C) Expression of GFP in *cyp-35B1p*::*gfp* was upregulated only when the worms were fed HT115. Images are of worms 48 hours post L4. Tails are marked by ‘T’. Images captured at 100x magnification. (D) Western blot analysis of WT or *flr-4(n2259)* grown on HT115 or OP50 using anti-phospho-PMK-1, anti-total PMK-1 or anti-β-actin antibodies. Quantification of the blot is shown in S9B Fig. Blots from 4 biological replicates available in S16 Fig. Day 1 adult worms were used for RNA-seq and QRT-PCR analysis.

Since *flr-4(n2259)* shows differential response to OP50 and HT115 to extend life span, we suspected that this may be due to the ability of a diet to upregulate a specific set of genes. So, we performed transcriptomic analysis of wild-type and the mutant on the two bacterial diets. We found that the XDP genes are upregulated only when *flr-4(n2259)* was grown on HT115 but not when grown on OP50 (Fig 5A). We verified several of these genes using qRT-PCR and found them to be upregulated only on HT115 (Figs 5B and S9A). Additionally, we used the *flr-4(n2259);cyp-35B1p*::*gfp* strain to show that the expression of GFP is induced only when the worms are grown on HT115 (Fig 5C). Together, this shows that *flr-4* mutant worms mount a specific p38-dependent transcriptional response when fed HT115 that provide cytoprotective benefits leading to enhanced life span.

Next, we asked whether only HT115 can differentially activate the p38 MAPK pathway. For this, we performed western blot analysis with WT and *flr-4(n2259)* grown on HT115 or OP50. Interestingly, we found that *flr-4(n2259)* grown on HT115 showed enhanced phosphorylation of PMK-1 compared to wild-type (Figs 5D, S9B and S15). However, the levels of phosphorylation were unchanged in WT maintained on the two bacteria. Thus, FLR-4 prevents differential activation of p38 MAPK dependent on diet, maintaining adaptive capacity in *C*. *elegans*.

### FLR-4 utilizes similar pathway as dietary restriction for longevity assurance

**Fig 6:**
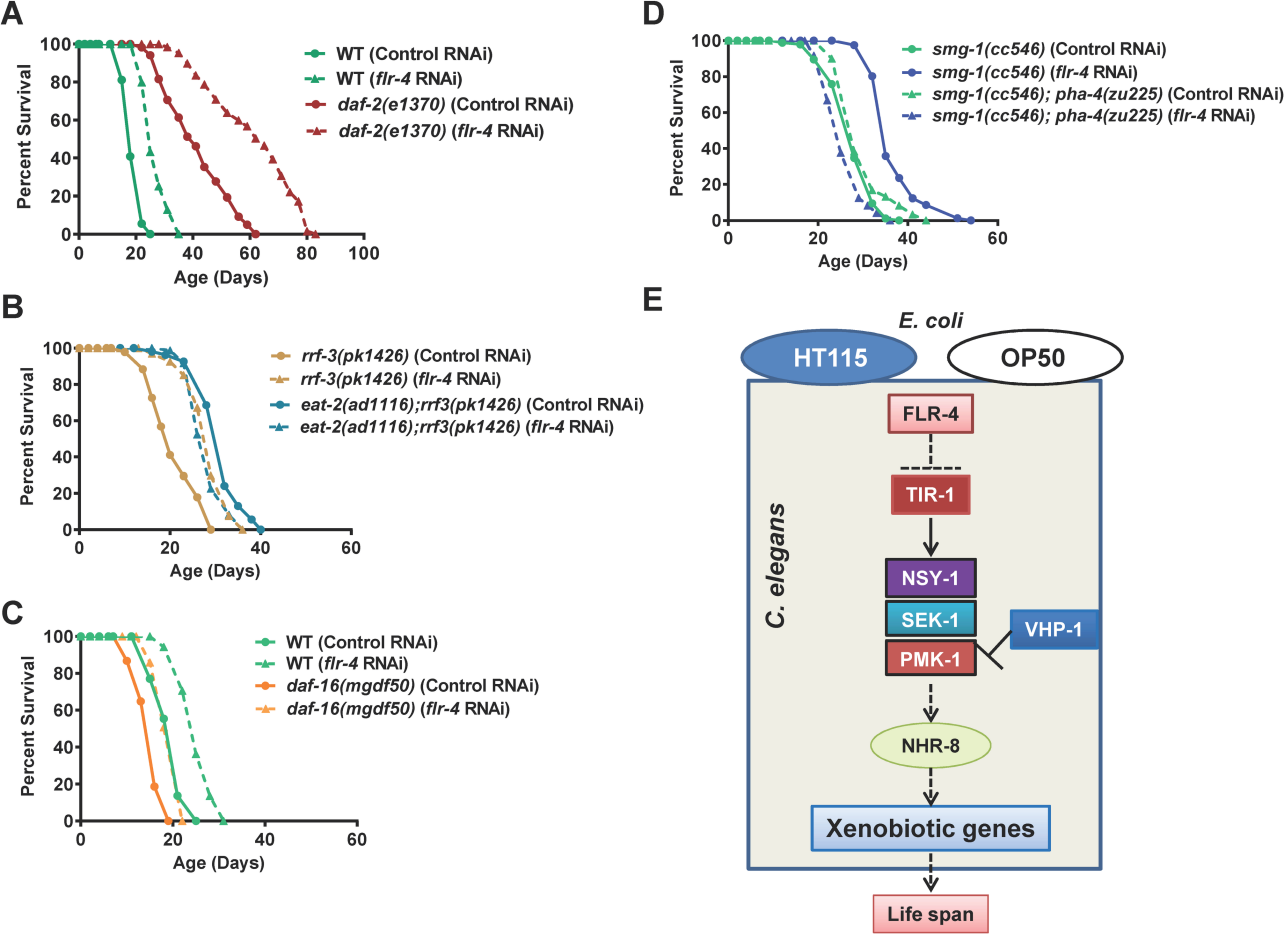
FLR-4 and dietary restriction use similar mechanisms for longevity assurance. (A) The life span of *daf-2(e1370)* was further increased when *flr-4* was knocked down using RNAi. (B) The life span of *eat-2(ad1116)* was not further extended when grown on *flr-4* RNAi. The RNAi hypersensitive strain *rrf-3(pk1426)* was used to ensure maximum RNAi efficiency in the feeding defective *eat-2* mutant. (C) The extended life span of *flr-4* RNAi worms is not dependent on *daf-16*. Life spans were performed at 20 ^o^C. (D) The life span extension on *flr-4* knockdown is dependent on *pha-4*. The *smg-1(cc546)* as well as *pha-4(zu225);smg-1(cc546)* were maintained at 25 ^o^C and the life span was performed at 15 ^o^C to inactivate PHA-4. (E) A model of FLR-4 function. FLR-4 negatively regulates the p38 MAPK pathway. RNAi knockdown or mutation in *flr-4* activates the pathway and leads to xenobiotic gene upregulation through NHR-8, providing life span benefits. In the kinase-dead *flr-4(n2259)* mutant, the pathway is only activated on HT115 and not OP50.

Since *flr-4* mutant worms responded differentially to diet, we evaluated the interaction of the gene with two nutrient sensing pathways. First, using RNAi we knocked down *flr-4* in the IIS pathway mutant *daf-2(e1370)* and found that the life span of the mutant is further extended, suggesting independent mechanisms (Fig 6A, S1 and S2 Tables). On the other hand, the life span of *eat-2(ad1116)* is not further extended; in fact, the life span was suppressed by 10–14% (Fig 6B). However, *flr-4* knockdown does not affect pharyngeal pumping of *eat-2(ad1116)*, similar to wild-type (S13 Fig), showing that the lack of additive effect on life span with *eat-2(ad1116)* is not mechanical. In case of another genetic mimic of DR [16], the extended life span of *drl-1* RNAi worms was also not extended further by *flr-4* mutation (S10A Fig). This suggested that *flr-4* uses cellular signalling pathways utilized by DR, but not the IIS pathway to ensure longevity. This was further supported by the fact that life span of *flr-4* RNAi, as in *eat-2* mutants and on *drl-1* knockdown, was dependent only on the FOXA transcription factor PHA-4, and not the FOXO factor DAF-16 that is required by IIS pathway mutants (Figs 6C, 6D and S10B). In the *pha-4(zu225)*, life span extension by *flr-4* knockdown was completely abrogated. Interestingly, the life span of the *flr-4(n2259)* or *flr-4* RNAi worms was independent of the NRF2 ortholog, SKN-1, a common output of insulin-like signalling and DR [36, 37](S10C and S10D Fig); *skn-1* abrogation by mutation or RNAi affects the life span of WT and *flr-4* knockdown worms to similar extent. Further, similar to DR [16, 38], the *flr-4* RNAi did not further extend the already long life span of germline defective mutants (S11 Fig). Like many long lived mutants, *flr-4* mutants have delayed reproductive span and lower brood size compared to WT, mainly on HT115 (S12 Fig). This may be due to more resource allocation towards somatic maintenance during DR [39] and may be caused by lower germ cell proliferation as seen in case of insulin-IGF-1 signalling pathway mutants [40]. The fact that FLR-4 utilizes the DR machinery for longevity assurance is also consistent with its role in ensuring adaptive capacity to diet.

## Discussion

A complex interaction between genes and diet determines the rate of aging and predisposes an individual to age-related diseases. The term gene-diet pair is used when the consequences of mutating a gene is visible only on a specific diet [12]. Surprisingly, only a few gene-diet pair have been identified that regulates aging, mainly through studies in *C*. *elegans* [12]. In this report, we identify a novel gene-diet pair and show that the adaptive capacity to different food-type is modulated by the protein kinase FLR-4. This protein prevents differential activation of the p38 MAPK pathway dependent on the food-type and consequently, the expression of cytoprotective genes by transcription factor NHR-8 (Fig 6E). Interestingly, this pathway overlaps with the DR pathway, suggesting a cross-talk between cellular signalling that senses food quality and quantity to regulate life history traits like aging.

FLR-4 is a serine-threonine protein kinase similar to mammalian Cyclin-dependent protein kinase 3 (31% identity, 50% similarity, E value 8e-22). It was initially identified in a screen for genes involved in fluoride tolerance [41], but was subsequently shown to have defects in ultradian rhythm in the intestine that controls defecation [15]. In this study, we elucidate a novel function for FLR-4 in the intestine and neurons that is independent of its role in defection. The temperature-sensitive kinase dead mutant *flr-4(n2259)* has normal defecation cycle at 20 ^o^C [15], a temperature at which most of our assays were performed. In view of the central role that this kinase plays in controlling multiple important phenotypes, future research needs to be directed towards finding its immediate cellular targets. This is particularly important as we found that the food-type-dependence is specific to the kinase dead mutant; RNAi knockdown using an OP50-based system also increased life span. This suggests that the kinase-dead mutant may not be able to phosphorylate a substrate(s) that is required to maintain life span homeostasis on the different bacterial diets.

Considering the importance of gene-diet pairs in aging and disease, our understanding of the mechanisms of adaptive capacity to food-type is still in its infancy. Previous studies have identified a few genes that play a role in this process. Notably among them are the RICTOR ortholog *rict-1* [6], neuromedin U receptor ortholog *nmur-1* [14] and mitochondrial 1-pyrroline-5-carboxylate dehydrogenase (P5CDH) *alh-6* [11]. The *rict-1* mutants have phenotypes similar to *flr-4* mutant worms such that they have shorter life span on OP50 while exhibiting life span extension on HT115 [6]. However, in contrast to the *flr-4*, *rict-1* regulates feeding behaviour when an animal encounters diets of different qualities. The *rict-1* mutants have different pumping rates and exhibit avoidance behaviour on food of diverse quality [6, 12]. The *alh-6* mutants, on the other hand, show accelerated aging when fed OP50 while retaining normal rates of aging on HT115 [11]. Interestingly, the *nmur-1* mutants have longer life span on OP50 but not on HT115 [14]. Thus, these gene-diet pairs seem to control diverse aspects of an animal’s response to different food. In future, it will be interesting to study the interaction of these genes with *flr-4*, considering the fact that opposing phenotypes controlled by these genes may indicate homeostatic control of life span in response to different diet.

As the above mutants differ in their response to different diet, they may also activate diverse signalling cascades. This is apparent from the fact that *rict-1* and *nmur-1* interact differentially with the downstream components of the insulin signalling pathway [6, 14, 42]. While the life span extension of *rict-1* knockdown is dependent on NRF2 ortholog *skn-1*, *nmur-1* mutants require the FOXO transcription factor DAF-16. We found that the *flr-4* mutants require the FOXA transcription factor PHA-4 for life span extension and is independent of DAF-16; its transcriptional response may thus be different from other gene-diet pairs. Since both *alh-6* and *flr-4* may signal through a pathway used by the *eat-2* model of DR, it will be interesting to study the transcription factor requirements of the former. Comparative gene expression profiles of these mutants on different diet will help us understand the complex gene expression modalities controlled by these gene-diet pairs.

Gustatory and olfactory neurons that perceive chemical signals have previously been shown to affect life span [10, 14, 43]. Here we show that *flr-4* knockdown in the neurons can also increase life span. In fact, the life span extension by *flr-4* RNAi requires the CAMKII ortholog UNC-43 and SARM ortholog TIR-1 that is known to act in the neurons to determine cell fate. On the other hand, intestine-specific knockdown of *flr-4* also increases life span. Although it appears intuitive to suggest that the gut may play an important role in sensing different diet that is ingested, the role of the intestine in food-type-dependent life span extension is less known. Interestingly, TORC2 that also regulates food-type-dependent life span, requires SKN-1/NRF2 in the intestine to regulate life span [42]. However, FLR-4 life span is independent on *skn-1*, indicating to extensive insulation as well as cross-talks among these pathways. Future research needs to be directed to understand the partitioning of the p38 MAPK pathway in the neurons and intestine as well as their cross-talk to regulate *flr-4*-mediated life span.

Animals in the wild, unlike those in laboratories, are exposed to a wide variety of food that they have adapted to. Being able to utilize a wide range of diet is evolutionarily advantageous as the animals can survive when their optimal diet is depleted. Giant panda that depend mainly on bamboo for nutrition is facing extinction due to loss of habitat (wwf.panda.org)[44]. Since diet influences the rate of aging, the animals have evolved intricate mechanisms to maintain homeostasis. In addition to the quality of diet, the quantity of food regulates the plasticity of aging. As a result, DR is able to delay aging and increase life span across the animal kingdom [45, 46]. In our study as well as in that of *alh-6* [11], we observe genetic overlap with DR, suggesting that organisms have evolved cellular modules that evaluate both quality and quantity of diet to regulate life span.

We show that FLR-4 signals through the p38 MAPK pathway to regulate the expression of cytoprotective genes dependent on diet. In *C*. *elegans*, this pathway has been extensively characterized for its role in mediating innate immune response towards pathogenic bacteria as well as in mounting an oxidative stress response [21, 23, 47–49]. On the other hand, the XDP genes have been shown to provide cytoprotective effects leading to enhanced life span in multiple models of longevity [16, 50–52]. The fact that FLR-4 would signal differences of diet through the p38 MAPK seems quite intuitive for an organism that feeds on bacteria and uses this same pathway to differentially activate immune genes on encountering pathogens. But in case of *flr-4* knock down, immune effector genes are not upregulated, showing specificity of this module. However, in mammals, the p38 MAPK has important role in regulating metabolism in liver and adipocytes during fasting, mediated by glucagon and insulin [53]. It will be interesting to study the role of p38 MAPK pathway in gene-diet interaction networks in mammals.

How *flr-4* mutants sense the differences in bacterial food remains to be answered. One possibility is that the mutants become sensitive to the presence or absence of a metabolite secreted by the bacteria and mount the specific response, whereas the WT worms are able to maintain homeostasis. Detailed metabolomics study will be able to reveal the exact nature of the molecule. One interesting observation is that genes that are upregulated in *flr-4(n2259)*, grown specifically on HT115, are enriched in amino acid metabolism. Previous studies have shown that methionine metabolism greatly influences life span, metabolism, and stress resistance [54–56]. Vitamin B12 acts an important cofactor in methionine as well as propionic acid metabolism, maintaining optimal level of Homocysteine and propionic acid, thereby preventing toxicity [54]. It is possible that the molecule may be vitamin B12, as seen in case of *Comamonas* [17, 57, 58]. In line with this idea, we found that the levels of the metabolic sensor *acdh-1* is much suppressed in HT115-fed *flr-4(n2259)* in our RNA-seq data, similar to the effect of vitamin B12 treatment. However, *flr-4* mutants do not have any significant defect in development or fat storage. In future, why *flr-4* mutants become sensitive to a metabolite or whether the two bacteria differ in production of soluble metabolites needs to be addressed. We also need to understand why the RNAi knockdown of the gene do not induce food-type-dependent life span response.

Together, our study discovers a new gene-diet pair that controls the plasticity of aging in *C*. *elegans* and reveals a complete signal transduction cascade involved in this process.

## Materials and methods

### *C*. *elegans* strains and maintenance

*C*. *elegans* strains used in this study were obtained from the Caenorhabditis Genetics Center and maintained on NGM agar plates at 20°C, unless otherwise stated, on *Escherichia coli* OP50 lawns. All RNAi experiments were initiated with synchronized L1 worms. Strains used in the study are: N2 Bristol as wild-type, *flr-4(n2259)X*, *rde-1(ne219) V*, *rde-1(ne219) V;kzIs9*, *rde-1(ne219) V;kzIs20*, *rde-1(ne213) V;kbIs7*, *sid-1(pk3321) V*, *sid-1(pk3321) V;uIs69 V*, *ccIs4251* [pSAK2 (*myo-3*::*NGFP-LacZ*)], *tir-1(tm3036)III*, *unc-43(e408)IV*, *nsy-1(ag3)II*, *nsy-1(ok593)II*, *sek-1(km4)X*, *pmk-1(km25)IV*, *pmk-3(ok169)IV*, *flr-4(n2259)X; sek-1(km4)X*, *nhr-8(ok186) IV*, *daf-2(e1370)III*, *bvIs5* [*cyp-35B1p*::*GFP + gcy-7p*::*GFP*] referred to as *cyp-35B1p*::*gfp* in this manuscript, *nhr-8(ok186) IV;bvIs5*, *flr-4(n2259)X;bvIs5*, *rrf-3(pk1426)II;eat-2(ad1116)II*, *rrf-3(pk1426)II*, *daf-16(mgdf50)I*, *smg-1(cc546)*, *smg-1(cc546)I;pha-4(zu225)V*, *skn-1(zu169) IV/nT1[unc-*?*(n754) let-*?*](IV;V)*, *glp-1(e2141)III*, *gld-1(op236)I*, *glp-4(bn2ts)I*, *hsf-1(sy441)*.

### Lifespan analysis

Gravid adult worms, initially grown on *E*. *coli* OP50, were bleached and the eggs were L1 synchronized in M9 buffer for 16 hours before placing them on the respective RNAi plates (say ‘X’ gene RNAi). Once worms reached L4 stage, they were transferred to intermediate RNAi plates (seeded with the same ‘X’ gene RNAi) for 12 hours. After that, the worms were picked onto fresh ‘X’ gene RNAi plates overlaid with 5-fluorodeoxyuridine (FUDR, final concentration 0.1 mg/ml of media). For life span analysis on plates without FUDR, worms were transferred to fresh plates on alternate days till the end of the reproductive span. Life span scoring was initiated at day 7 of adulthood and continued every alternate day. For statistical analyses of survival, OASIS software (http://sbi.postech.ac.kr/oasis) was used and *P*-values were calculated by using a log rank (Mantel-Cox method) test.

For temporal requirement experiments, L1 synchronized worms were placed on control RNAi plates. Worms from the plates were transferred to *flr-4* RNAi plates at L2, L3, L4 or YA stages. FUDR was overlaid on the plates 12 hrs after the worms reached L4.

For life span on different bacterial feed, L1 synchronized worms were placed on HT115 and OP50-seeded plates and the lifespan was initiated as mentioned above.

All life span analysis referred in the main text is provided in the S1 Table. Two independent biological replicates are provided in S2 Table.

### Body bend assay

A total of 20 worms each from control or *flr-4* RNAi plates were transferred to an unseeded plate on day 2, 5 and 10 of adulthood. Each worm was gently prodded on the tail with a platinum wire and total number of body bends per 30 seconds was counted. A body bend was scored every time the area behind the pharynx reached a maximum bend in the opposite direction from the last bend counted.

### Muscle integrity measurements

The *ccIs4251* [pSAK2 (*myo-3*::*NGFP-LacZ*)] worms were grown on control or *flr-4* RNAi plates. On day 2, 5 and 10 of adulthood, the worms were paralyzed on 2% agarose pads in the presence of 20 mM sodium azide. Photographs of the worms were captured at 630X magnification using an AxioImager M2 microscope (Carl Zeiss, Germany) fitted with Axiocam MRm [Excitation 488nm and Absorbance at 520nm]. For each RNAi, at least 10 nuclei of 10 worms each were photographed. Morphology of each muscle nuclei was scored as ‘intact’, ‘moderately damaged’ or ‘severely damaged’. A nucleus was scored as ‘intact’ if it had intact membrane with no degradation, ‘moderately damaged’ when the nuclear membrane appeared to disintegrate but the nucleoplasm displayed no or very little dark patches and ‘severely damaged’ when the nuclei had increased nucleolar size, dark patches in the nucleoplasm, distorted appearance and membrane disintegration.

### Lipofuscin autofluorescence

To determine lipofuscin autofluorescence, 20 worms each grown on control or *flr-4* RNAi were anesthetized in 20mM Sodium Azide and mounted on 2% agarose pads on day 1, 5 and 10 of adulthood. The worms were visualized under microscope using FITC filter and images were captured using a constant exposure time (1.2 sec).

### RFP beads ingestion assay

Twenty five L4 worms, grown on respective bacterial feed, were picked and placed onto NGM plates seeded with 250:1 (vol:vol) of bacteria and Fluoresbrites Multifluorescent microspheres/RFP beads (0.2 μm diameter, Polyscience Inc., USA). After 10 minutes, worms were collected and washed twice with 1X M9 buffer to remove any bead attached to the body surface. Worms were finally re-suspended in 30 μl of 1X M9 buffer and transferred to a freshly prepared 2% agarose pad slides. The images of worms were taken using AxioImager M2 microscope (Carl Zeiss, Germany). Quantification was performed using NIH ImageJ software.

### Pharyngeal pumping rate

An one minute video of Day 1 adult worms was taken using Axiocam MRm camera attached to M205FA microscope (Leica, Germany). The video was slowed down and pharyngeal pumping was counted for that 10 second period.

### Body size

Worms were imaged one day after they reached L4 using Axiocam MRm camera attached to an AxioImager M2 microscope (Carl Zeiss, Germany). Area of the worms was quantified using NIH ImageJ software.

### Cloning

*flr-4* RNAi: The full length cDNA sequence of *flr-4* was amplified using primers listed in S3 Table and cloned into pL4440 RNAi vector.

### Generation of *flr-4* transgenic worms

A transcriptional fusion of the *flr-4* promoter and a green fluorescent protein (GFP) gene was constructed in pPD95.75. The 3.5 kb promoter region upstream of start codon of *F09B12*.*6* was amplified using primers listed in S3 Table and HiFidelity PCR system (Kapa Biosystems, USA) and cloned into pPD95.75 using *Bam*HI and *Kpn*I restriction sites. The recombinant plasmid was injected at a concentration of 5ng/μl into the syncytial gonad of wild-type worms along with 100ng/μl pRF4 (*rol-6)* co-injection marker using a Microinjection setup consisting of Nikon TiS inverted microscope fitted with Eppendorf Femtojet Express and Transferman NK2. Transformants were selected based on the rolling phenotype as well as the presence of GFP expression. Fluorescence images of transgenic worms were captured under AxioImager M2 microscope (Carl Zeiss, Germany) fitted with Axiocam MRm at 40X magnification [Excitation 488nm and Absorbance at 520nm].

The full length cDNA sequence of *flr-4* was amplified using primers listed in S3 Table. The *gfp* sequence of *Pflr-4*::*gfp* plasmid was then excised using *Kpn*I and *Eco*RI and replaced with the amplified *flr-4* cDNA sequence, generating the *Pflr-4*::*flr-4 cDNA* construct. The *Pflr-4*::*flr-4* cDNA construct and pRF4 were co-injected in the germline of *flr-4(n2259)* (concentrations: 150 ng/μL pRF4 and 5 ng/μL *Pflr-4*::*flr-4 cDNA*). Wild-type and *flr-4(n2259)* roller lines were generated by injecting 150 ng/μL pRF4. Lines were maintained by picking rollers.

### RNA isolation

Synchronized L1 worms grown on OP50 or RNAi plates were collected at Day 1 of adulthood in M9 buffer and washed thrice using M9 buffer. Then, Trizol was added to about 4 times the volume of the worm pellet and the worms lysed using two freeze thaw cycles, followed by vigorous vortexing. RNA was purified by phenol:chloroform:isoamylalcohol extraction and isopropanol precipitation. For quantitative Reverse Transcriptase PCR (qRT-PCR) experiments, the concentrations of the RNA were determined using NanoDrop 2000 (Thermo Scientific, USA) and the quality of the ribosomal 28 S and 18 S on denaturing agarose gel was used for evaluation of RNA integrity. For transcriptomic analysis, the quality was evaluated using Bioanalyzer (Agilent, USA) and only RNA with RIN number above 9 was used for RNA-seq.

### QRT-PCR analysis

About 2.5 μg of RNA was converted to cDNA using Superscript III Reverse Transcriptase enzyme and poly-T primers (Invitrogen, USA). QRT-PCR analysis was performed using the DyNAmo Flash SYBR Green mastermix (Thermo Scientific, USA) and Realplex PCR system (Eppendorf, USA) to determine the relative gene expression levels. Statistical analysis was performed using GraphPad 7.0. All the primers used are listed in S3 Table.

### Transcriptomic analysis

RNA-Sequencing (RNA-seq) libraries of WT grown on Control RNAi or *flr-4* RNAi, and WT or *flr-4(n2259)* grown on HT115 or OP50 at Day 1 adulthood were prepared as recommended by the Illumina TruSeq RNA Sample Preparation kit using Low-Throughput (LT) Protocol (Illumina, Inc., USA). Sequencing of libraries was performed using Illumina GA*II*_*X*_ for 78 cycles including 6 additional cycles for index read. Sequence reads were aligned using CLC Genomics Workbench 6.5.1 with default setting against *C*. *elegans* genome assembly (WS231). Unpaired group comparisons, based on RPKM (Reads per Kilobase per Million mapped reads), were chosen as expression values for comparing the samples. A fold change ±2.0 and *P* value ≤0.05 (Kal's Z test) were used to filter the differentially expressed genes. GO‐term enrichment analysis was performed using the DAVID Bioinformatics Database [32]. The sequencing data is available as BioProject ID: PRJNA362992.

### Western blotting

Synchronized L1 worms, grown on OP50 or HT115-seeded plates, were collected at Day 1 of adulthood in 1xM9 buffer and washed thrice using the same buffer. The pellet was freeze-thawed 3 times in a protein extraction buffer (20 mM Hepes buffer pH 7.9, 25% glycerol, 0.42 mM NaCl, 1.5 mM MgCl_2_ hexahydrate, 0.2 mM EDTA dihydrate, 0.5 mM DTT) in presence of a protease inhibitor cocktail (Sigma, USA), sonicated in a waterbath-based sonicator (Diagenode, USA) and centrifuged at 10,000 rpm for 10 mins. The protein concentration in the supernatant was estimated by using Bradford reagent (BioRad, USA).

About 30 μg of protein was separated on a 12% SDS-PAGE and transferred to Nitrocellulose membrane. The membranes were blocked for 1 hour in 5% non-fat milk and 5% BSA dissolved in 1X TBST (TBS with 0.1% Tween 20) and probed with anti-PMK-1 antibody (1:2,000 dilution in blocking buffer; Cell Signaling Technology, USA) or anti-phospho-PMK-1 antibody (1:2,000 dilution in blocking buffer; Cell Signaling Technology, USA), incubated overnight at 4°C. Next day, the membranes were washed thrice with 1X TBST and further incubated with 1:10,000-diluted secondary antibody (anti-rabbit conjugated to HRP, Cell Signaling Technology, USA) for 1 hr at room temperature. The blots were then washed 4–5 times with 1X TBST, each wash lasting 10 min. The blots were developed using enhanced chemiluminiscent substrate (Millipore, USA).

For the quantification of PMK-1 activity, the band intensities of pPMK-1 and total PMK-1 were quantified using ImageJ software (National Institutes of Health, Bethesda, MD; http://rsb.info.nih.gov/ij/) and divided with the intensity of the beta-actin bands. The value thus acquired for pPMK-1 was then divided by that of total PMK-1 and represented as percentage. The immunoblots of four independent experiments were quantified.

### Arsenite treatment

Well-fed young adult worms from control RNAi-seeded plates were collected and washed thrice in M9 buffer. The worm pellet was then divided into two halves; to one half 120 μl of 1X M9 buffer was added while to the other half 120 μl of 1X M9 buffer containing 20mM sodium arsenite was added. After incubation at 20°C for 20 minutes, the worms were washed thrice with M9 buffer. The worm pellet was then processed for protein isolation and western blotting using the above-mentioned method.

### UV-resistance assay

Worms were grown on respective RNAi from L1 onwards. For each RNAi, four 60 mm unseeded NGM plates with approximately 25 L4 worms per plate were irradiated using a 254 nm UV bulb at 10Jm^-2^min^-1^ in a CL-1000 UV Crosslinker (Ultra-Violet Products Limited), followed by transfer to the respective RNAi-seeded NGM. All UV-resistance assays were performed at 20 ^o^C. Survival to stress was scored every 24 hrs post UV exposure.

### Heat-resistance assay

The worms were grown on RNAi plate as above. For each RNAi, three 60 mm NGM plates with approximately 40 L4 worms per plate were incubated at 35 ^o^C. Animal survival was scored every 60 min.

### Reproductive span and brood size assay

Wild-type or *flr-4(n2259)* mutant worms were grown on two different *E*. *coli* feed, OP50 or HT115 till late L4 stage. Five worms were picked onto fresh plates (OP50 or HT115 seeded) and allowed to lay eggs for 24 hours. Three such plates were used for each assay so that ‘n’ was 15 per experiment. The worms were then transferred to fresh plates every day and the eggs/L1s on previous day’s plate were counted. Worms that crawled off the plates or ruptured before the fertile period ended were discarded. Eggs that produced viable progeny were considered as total L1s and the un-hatched eggs were considered as dead eggs. Pool of total L1s and dead eggs are defined as brood size. Data is presented as brood size ± SEM. For calculating reproductive span, total number of L1s is expressed per worm per day. Data is shown as viable progenies plotted against number of days, with SEM at each time point.

### Development rate

Gravid adult worms, initially grown on *E*. *coli* OP50, were bleached and the eggs were L1 synchronized in M9 buffer for 16 hours before placing them on seeded NGM plates. The worms were then scored every 12 hours till 60^th^ hour for their development stage.

### Ethics statement

The study was performed with approval from the Institutional Biosafety committee. Only invertebrate nematodes were used for the study.

## Supporting information

S1 Fig (A) Life span of WT worms is extended to same extent in absence or presence of FUDR, when *flr-4* is knocked down using RNAi. In absence of FUDR, worms were transferred to fresh plates every day during the reproductive phase. (B) The *flr-4(n2259)* worms have increased life span when grown on HT115 compared to OP50, both in absence as well as in presence of FUDR. Life spans performed at 20 ^o^C.
(PDF)

S2 Fig (A) The *flr-4* RNAi worms have lower lipofuscin accumulation with age as compared to age-matched WT worms. Images captured at 100x magnification. (B) Muscle nuclei degeneration was delayed in *myo-3*::*gfp* transgenic worms grown on *flr-4* RNAi. Nuclei were categorized as intact, moderately or severely degraded according to representative photographs in the left (refer to materials and methods). n > 20. Images captured at 630x magnification. (C) Analysis of age-dependent changes in number of body bends in worms grown on control or *flr-4* RNAi. Student’s *t* test was used to determine statistical significance on each day between control and *flr-4* RNAi-treated worms, n>40. (D) The *flr-4* RNAi worms have smaller body size compared to control RNAi. (E) Pharyngeal pumping does not change significantly when day 1 adult WT or *flr-4(n2259)* was grown on either HT115 or OP50. Average of 6 biological replicates shown. In each replicate, >15 worms were monitored. (F) WT as well as *flr-4(n2259)* worms ingest similar amounts of RFP-tagged beads when maintained on HT115 or OP50. For analysis of body size and RFP beads ingestion, Day 1 adult worms were used.
Error bars indicate SEM. ****P≤0.0001, **P≤0.01,*P≤0.05, n.s. is not significant. Student’s t-test.
(PDF)

S3 Fig Developmental rate of WT and *flr-4(n2259)* worms when maintained on control RNAi or flr-4 RNAi. Worms were synchronized at L1 and stages were determined at the indicated hours. *P*-value not significant in all except at 60^th^ hour between WT and *flr-4(n2259)* on control RNAi (Two-way ANOVA).
(PDF)

S4 Fig (A) UV stress tolerance assay was performed with WT worms grown on control or *flr-4* RNAi. The L4 stage worms were exposed to 10 J m^-2^ min^-1^ of UV and mortality was scored every day. (B) Heat stress assay was performed by exposing the L4 stage WT worms grown on control or *flr-4* RNAi to 35 ^o^C and mortality scored every hour. (C) The life span of WT and *hsf-1(sy441)* are extended to the similar extent when *flr-4* is knocked down. (D) *Hsf-1* RNAi suppressed the life span of WT and *flr-4(n2259)* to similar extent. Life span performed at 15 ^o^C.
(PDF)

S5 Fig Developmental rate of WT and *flr-4(n2259)* worms when maintained on HT115 or OP50. Worms were synchronized at L1 and stages were determined at the indicated hours. *P-*value not significant (Two-way ANOVA).
(PDF)

S6 Fig *Flr-4* knockdown is required early in life to increase life span. Maximum life span is observed when *flr-4* is knocked down starting at L1 or L2 (A-B). The effect decreases when knockdown is initiated at L3 or L4 (C-D). No life span increase was observed when knockdown was initiated on day 1 of adulthood (E). All life spans were performed at 20 ^o^C.
(PDF)

S7 Fig (A) The extended life span of *flr-4(n2259)* is suppressed by *sek-1* RNAi. (B) The life span of *flr-4(n2259)* is suppressed to the levels of *sek-1(km4)* in the double mutant *flr-4(n2259);sek-1(km4)*. Life spans performed at 20 ^o^C. (C) Western blot analysis of day 1 adult WT, *unc-43(e408)* or *tir-1(tm3036)* grown on control or *flr-4* RNAi using anti-phospho-PMK-1, anti-total PMK-1 or anti-β-actin antibodies. Quantification of the blot is shown below. The intensity of pPMK-1 and PMK-1 bands were normalized to beta-actin bands. Percent intensity of pPMK-1 with respect to (w.r.t.) total PMK is plotted. Average of 3 experiments shown. Error bars are SEM. **P≤0.01, n.s. not significant, Student’s *t* test. The activation of p38 MAPK pathway with 20 mM Arsenite was used as a control.
(PDF)

S8 Fig (A) Fold changes based on RPKM values between WT on control or *flr-4* RNAi as determined by RNA-seq. (B) The expression of *ugt-16* and *ugt-18* are upregulated when *flr-4* is knocked down using RNAi. (C) The expression of GFP in *cyp-35B1p*::*gfp* was induced in the lower gut region when *flr-4* is knocked down using RNAi. Head and tail are marked with H and T, respectively. Images are of worms 48 hours post L4. Images captured at 100x magnification. (D) The expression of *ugt-16* and *ugt-18* are not upregulated in *sek-1(km4)* to the same extent as in WT, when *flr-4* is knocked down using RNAi. (E) The life span of *flr-4(n2259)* is suppressed to a greater extent (35% against 21%) compared to WT when these worms were grown on *nhr-8* RNAi. (F) The expression of *ugt-18*, *cyp-32B1* and *gst-28* are not upregulated in *nhr-8(ok186)* to the same extent as in WT, when *flr-4* is knocked down using RNAi. Error bar indicates SEM. ****P≤0.0001, ***P≤0.001, **P≤0.01, *P≤0.05, n.s. not significant, Student’s *t* test. Day 1 adult worms were used for RNA-seq and QRT-PCR. (G) The life span of WT worms does not change when *vhp-1* is knocked down using RNAi. Life spans were performed at 20 ^o^C.
(PDF)

S9 Fig (A) QRT-PCR validation of RNA-seq data for *ugt-16* which is upregulated only when *flr-4(n2259)* worms were fed HT115. (B) Quantitation of data of Fig 5D. The intensity of pPMK-1 and PMK-1 bands were normalized to beta-actin bands. Percent intensity of pPMK-1 with respect to (w.r.t.) total PMK is plotted. Average of four experiments shown. Error bars are SEM. ***P≤0.001, **P≤0.01, *P≤0.05, n.s. not significant, Student’s *t* test. Day 1 adult worms were used for QRT-PCR and western blot analysis.
(PDF)

S10 Fig (A) Knocking down *drl-1* by RNAi does not further prolong the extended life span of *flr-4(n2259)*. (B) The *daf-16* RNAi suppresses life span of WT and *flr-4(n2259)* to similar extent. (C) The *skn-1* RNAi suppresses life span of WT and *flr-4(n2259)* to similar extent. (D) The *flr-4* RNAi extends life span of WT and *skn-1(zu169)* to similar extent. Life spans were performed at 20 ^o^C.
(PDF)

S11 Fig (A-C) Life span analysis was performed on different germline-defective mutants that were grown on control or *flr-4* RNAi. The worms were maintained at 15 ^o^C and life spans performed at 25 ^o^C.
(PDF)

S12 Fig (A) Reproductive span analysis of WT or *flr-4(n2259)* grown on HT115 or OP50. Number of eggs that hatched are plotted against the number of days. (B) Total number of eggs hatched over the entire reproductive span in shown. Comparisons are made between the WT and mutant on a particular diet. Error bars are SEM. ***P≤0.001, **P≤0.01, *P≤0.05, n.s. not significant, Student’s t test.
(PDF)

S13 Fig Pharyngeal pumping of wild-type or *eat-2(ad1116)* on control or *flr-4* RNAi. Error bars are SEM. **P≤0.01, n.s. not significant, Student’s t test.
(PDF)

S14 Fig All four biological replicates that were used for quantification of data is shown. Refers to Fig 3B.
(PDF)

S15 Fig All four biological replicates that were used for quantification of data is shown. Refers to Figs 5D and S9B.
(PDF)

S16 Fig Rescuing *flr-4*(n2259) with a wild-type copy of *flr-4* cDNA suppresses life span to the level of WT. Transgenic worms were generated by injecting WT and *flr-4(n2259)* with pRF4 (*rol-6*) plasmid as well as *flr-4(n2259)* with *Pflr-4*::*flr-4* cDNA along with pRF4. Life spans performed at 20 ^o^C.
(PDF)

S1 Table Life span details reported in the main text. (XLSX)

S2 Table Two representative sets of biological replicates for the life spans reported in the main text. (XLSX)

S3 Table Details of primers used in this study. (XLSX)
