# Supplementary figures and images for: A novel gene-diet pair modulates C. elegans aging
Source: PLoS Genet. 2018 Aug 20;14(8):e1007608. doi: 10.1371/journal.pgen.1007608 (PMC6117094; doi:10.1371/journal.pgen.1007608)

Figure S1

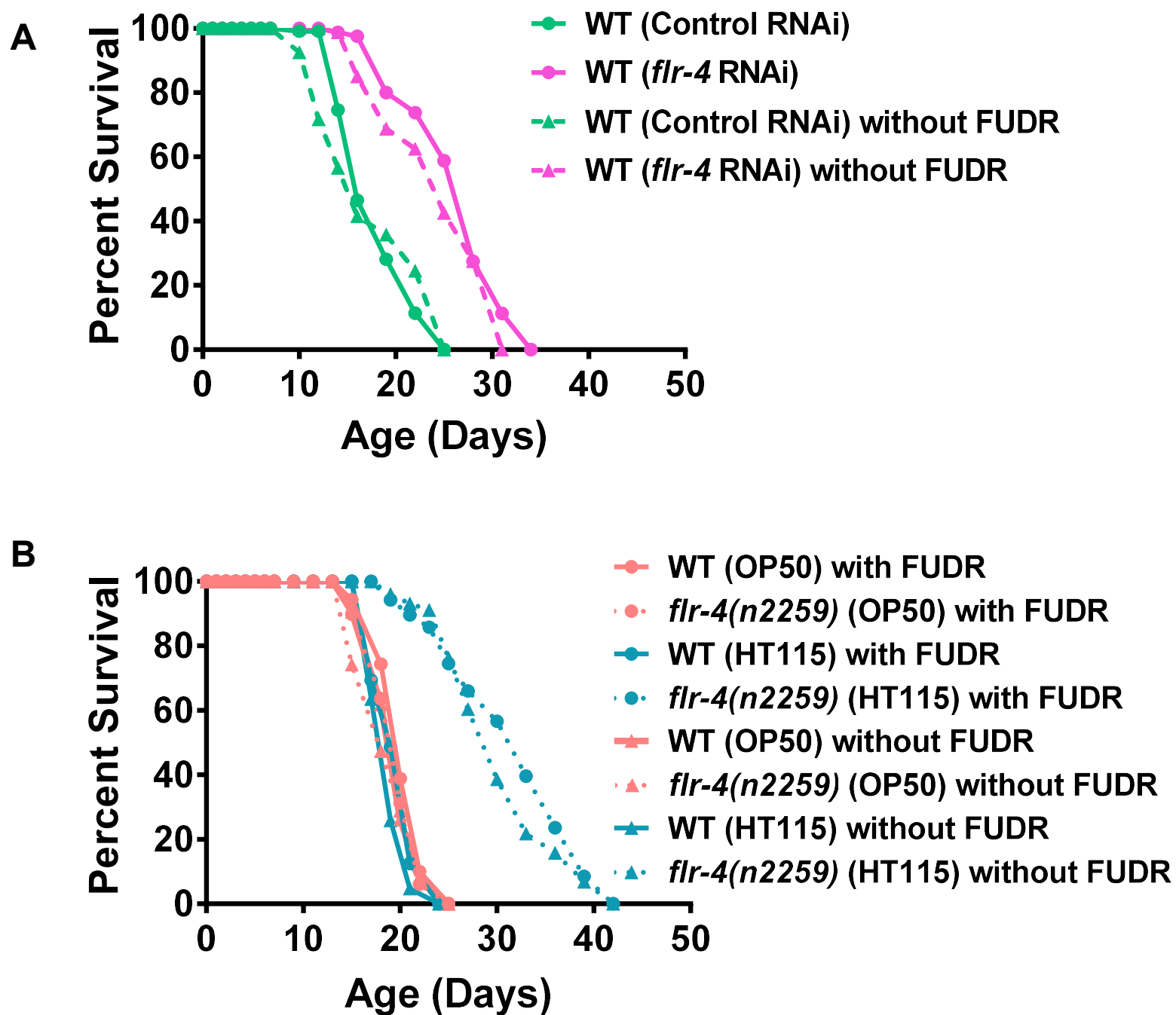

Supplement: S1 Fig — (A) Life span of WT worms is extended to same extent in absence or presence of FUDR, when flr-4 is knocked down using RNAi. In absence of FUDR, worms were transferred to fresh plates every day during the reproductive phase. (B) The flr-4(n2259) worms have increased life span when grown on HT115 compared to OP50, both in absence as well as in presence of FUDR. Life spans performed at 20 oC. (PDF) [file pgen.1007608.s001.pdf]

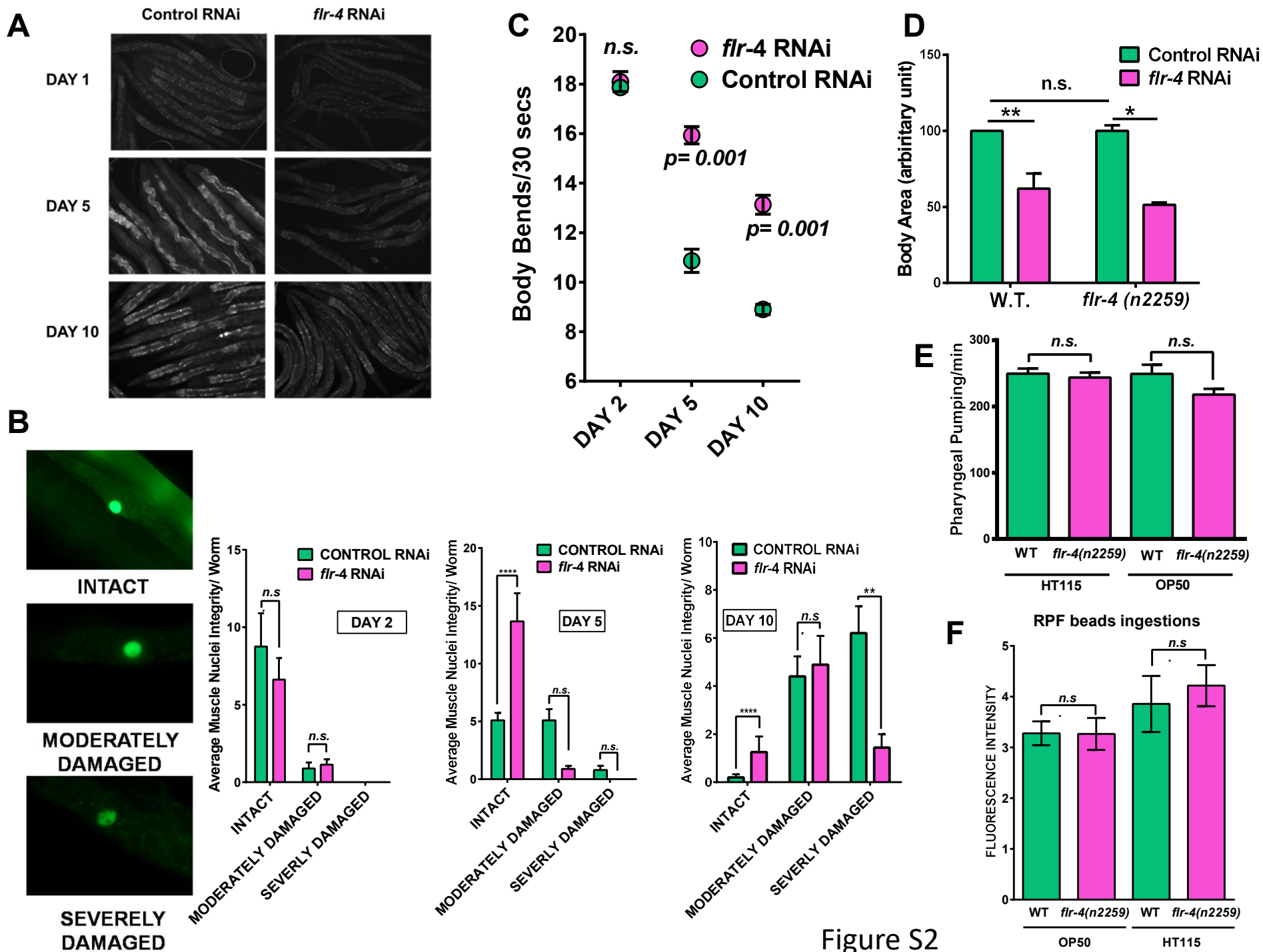

Figure S2

Supplement: S2 Fig — (A) The flr-4 RNAi worms have lower lipofuscin accumulation with age as compared to age-matched WT worms. Images captured at 100x magnification. (B) Muscle nuclei degeneration was delayed in myo-3::gfp transgenic worms grown on flr-4 RNAi. Nuclei were categorized as intact, moderately or severely degraded according to representative photographs in the left (refer to materials and methods). n > 20. Images captured at 630x magnification. (C) Analysis of age-dependent changes in number of body bends in worms grown on control or flr-4 RNAi. Student’s t test was used to determine statistical significance on each day between control and flr-4 RNAi-treated worms, n>40. (D) The flr-4 RNAi worms have smaller body size compared to control RNAi. (E) Pharyngeal pumping does not change significantly when day 1 adult WT or flr-4(n2259) was grown on either HT115 or OP50. Average of 6 biological replicates shown. In each replicate, >15 worms were monitored. (F) WT as well as flr-4(n2259) worms ingest similar amounts of RFP-tagged beads when maintained on HT115 or OP50. For analysis of body size and RFP beads ingestion, Day 1 adult worms were used. Error bars indicate SEM. ****P≤0.0001, **P≤0.01,*P≤0.05, n.s. is not significant. Student’s t-test. (PDF) [file pgen.1007608.s002.pdf]

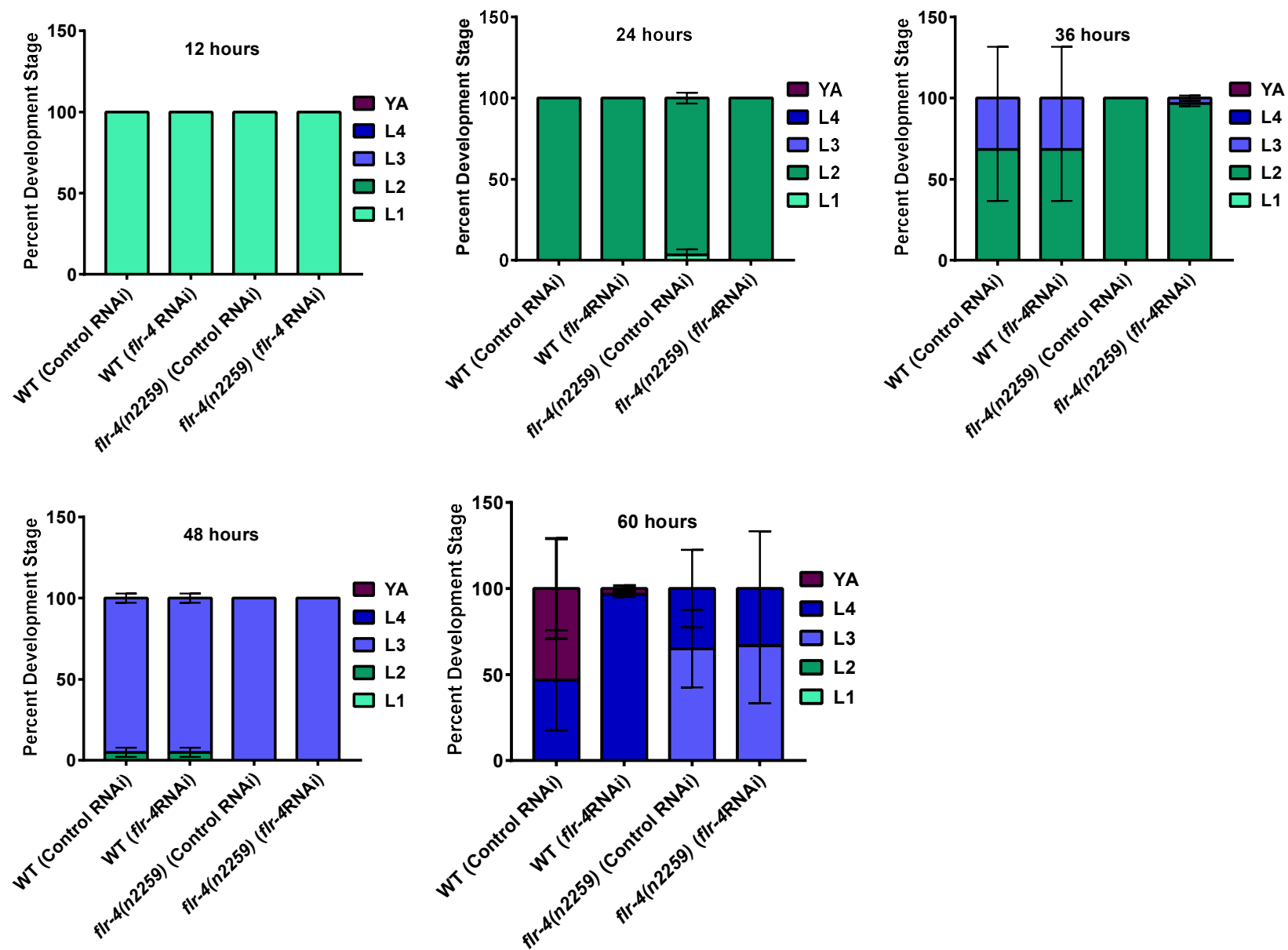

Figure S3

Supplement: S3 Fig — Worms were synchronized at L1 and stages were determined at the indicated hours. P-value not significant in all except at 60th hour between WT and flr-4(n2259) on control RNAi (Two-way ANOVA). (PDF) [file pgen.1007608.s003.pdf]

Figure S4

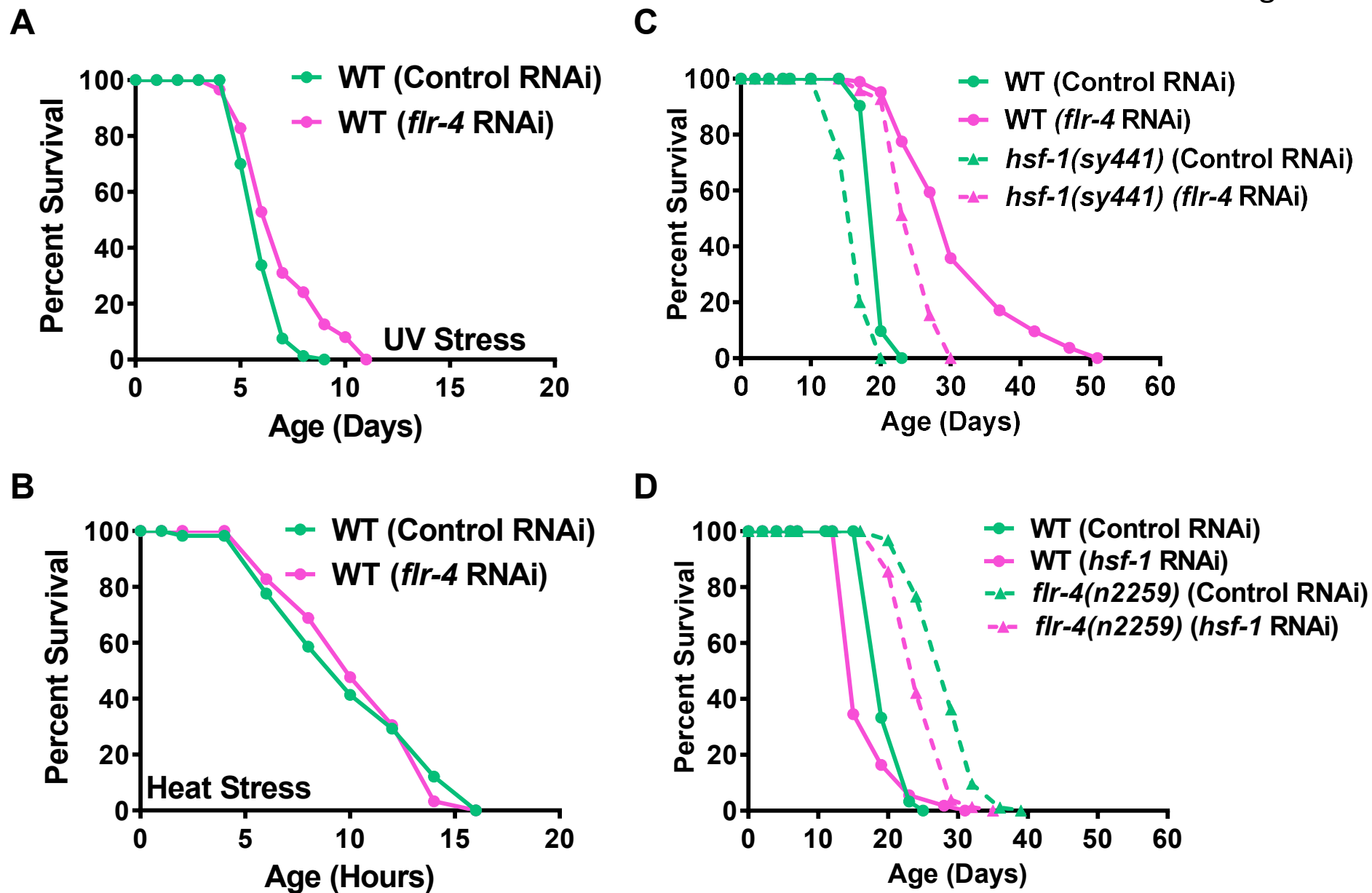

Supplement: S4 Fig — (A) UV stress tolerance assay was performed with WT worms grown on control or flr-4 RNAi. The L4 stage worms were exposed to 10 J m-2 min-1 of UV and mortality was scored every day. (B) Heat stress assay was performed by exposing the L4 stage WT worms grown on control or flr-4 RNAi to 35 oC and mortality scored every hour. (C) The life span of WT and hsf-1(sy441) are extended to the similar extent when flr-4 is knocked down. (D) Hsf-1 RNAi suppressed the life span of WT and flr-4(n2259) to similar extent. Life span performed at 15 oC. (PDF) [file pgen.1007608.s004.pdf]

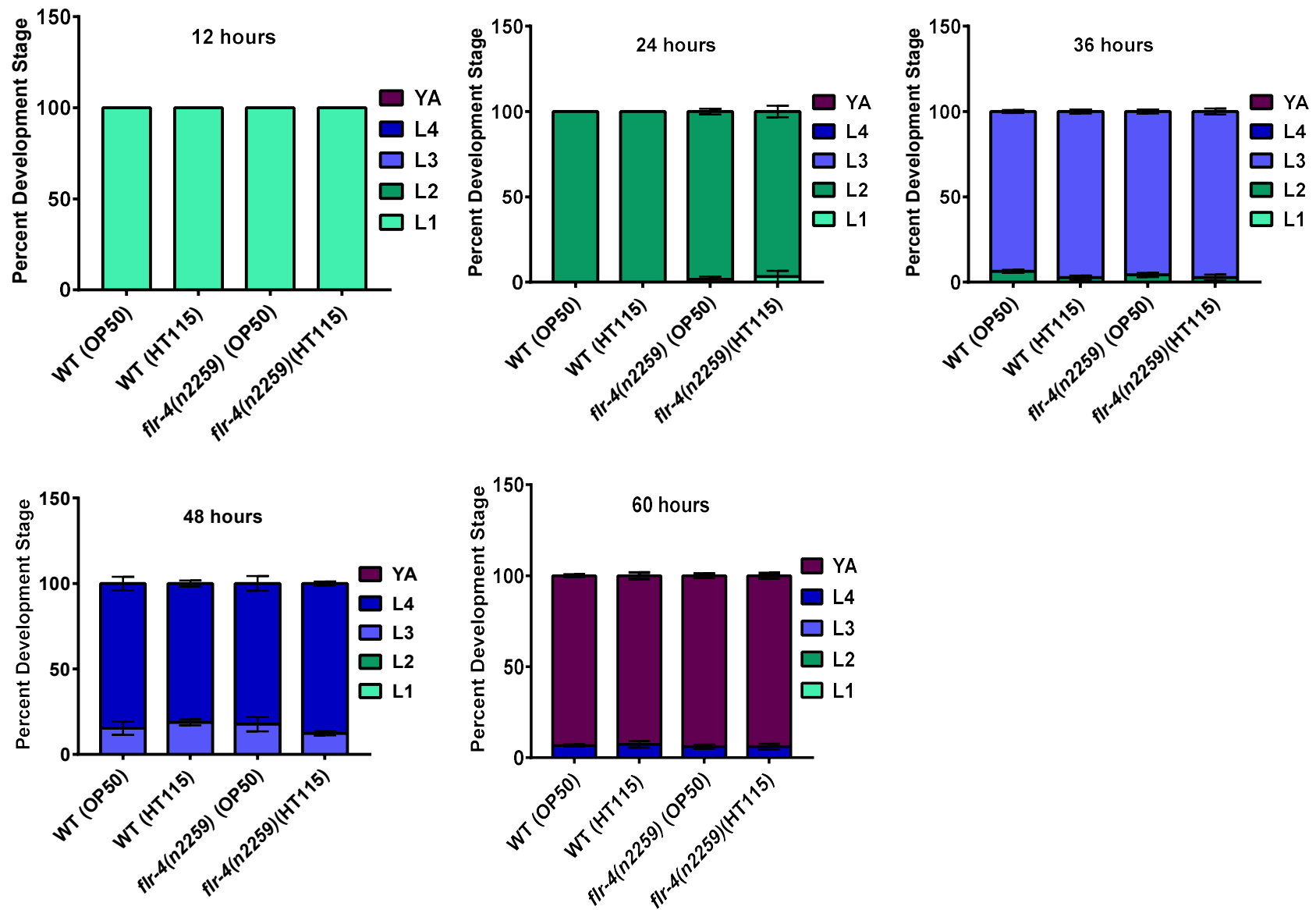

Figure S5

Supplement: S5 Fig — Worms were synchronized at L1 and stages were determined at the indicated hours. P-value not significant (Two-way ANOVA). (PDF) [file pgen.1007608.s005.pdf]

Figure S6

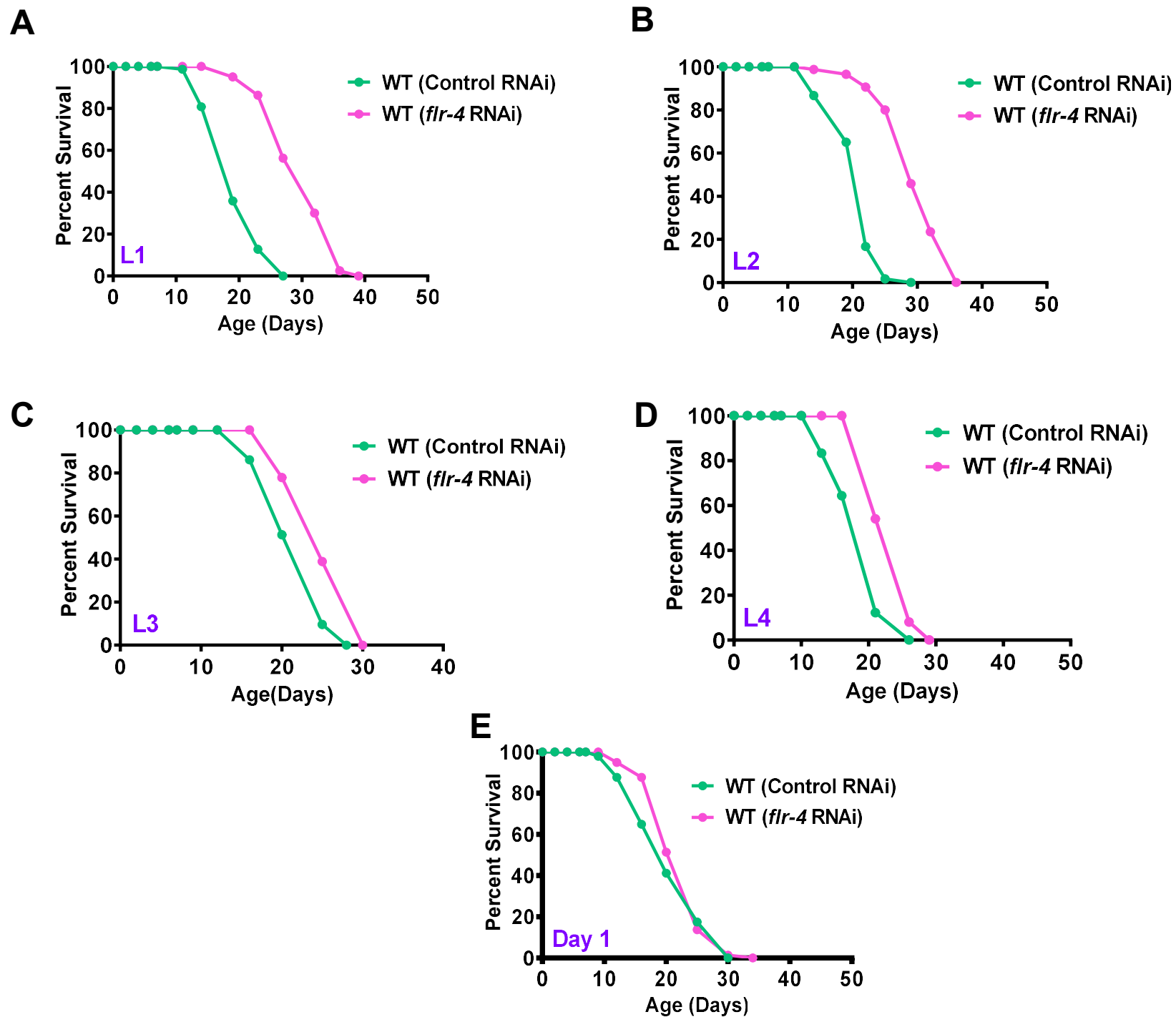

Supplement: S6 Fig — Maximum life span is observed when flr-4 is knocked down starting at L1 or L2 (A-B). The effect decreases when knockdown is initiated at L3 or L4 (C-D). No life span increase was observed when knockdown was initiated on day 1 of adulthood (E). All life spans were performed at 20 oC. (PDF) [file pgen.1007608.s006.pdf]

**A**

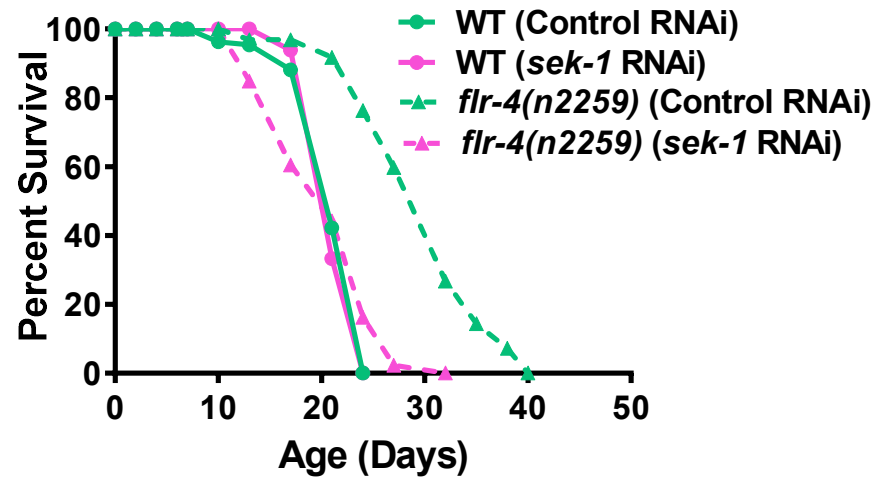

**B**

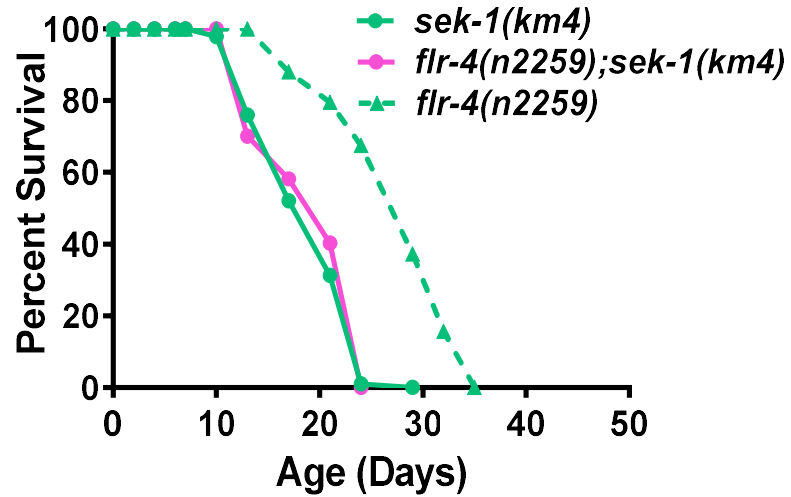

**C**

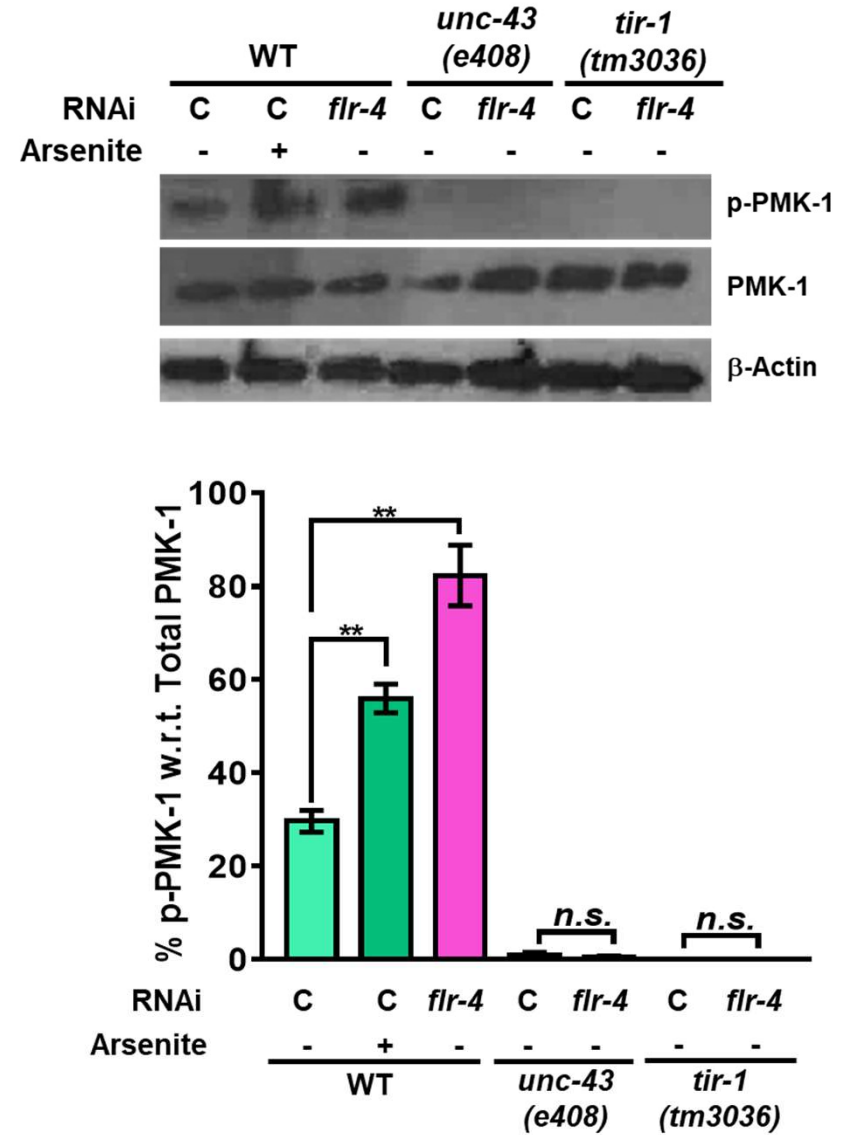

Figure S7

Supplement: S7 Fig — (A) The extended life span of flr-4(n2259) is suppressed by sek-1 RNAi. (B) The life span of flr-4(n2259) is suppressed to the levels of sek-1(km4) in the double mutant flr-4(n2259);sek-1(km4). Life spans performed at 20 oC. (C) Western blot analysis of day 1 adult WT, unc-43(e408) or tir-1(tm3036) grown on control or flr-4 RNAi using anti-phospho-PMK-1, anti-total PMK-1 or anti-β-actin antibodies. Quantification of the blot is shown below. The intensity of pPMK-1 and PMK-1 bands were normalized to beta-actin bands. Percent intensity of pPMK-1 with respect to (w.r.t.) total PMK is plotted. Average of 3 experiments shown. Error bars are SEM. **P≤0.01, n.s. not significant, Student’s t test. The activation of p38 MAPK pathway with 20 mM Arsenite was used as a control. (PDF) [file pgen.1007608.s007.pdf]

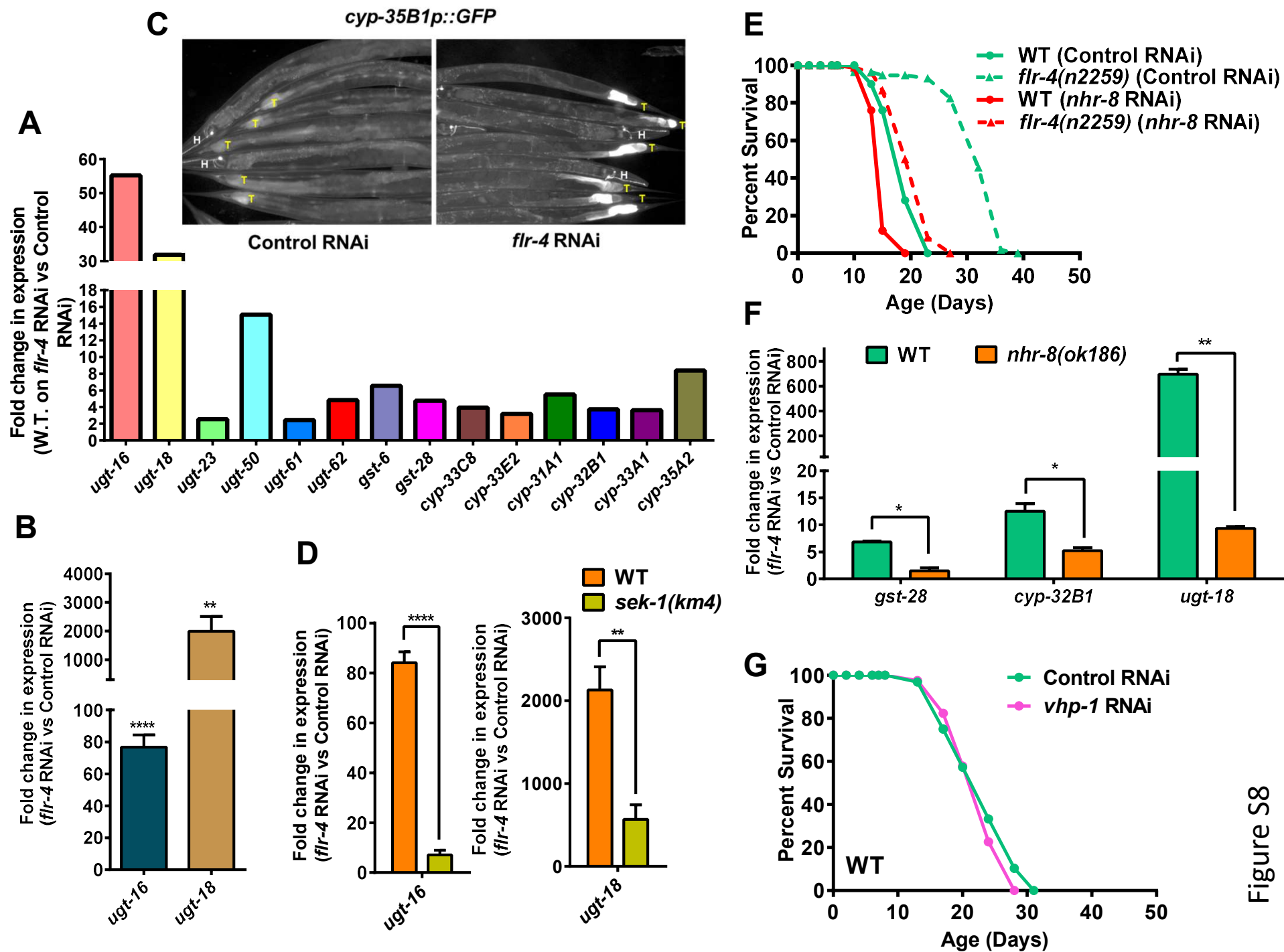

Figure S8

Supplement: S8 Fig — (A) Fold changes based on RPKM values between WT on control or flr-4 RNAi as determined by RNA-seq. (B) The expression of ugt-16 and ugt-18 are upregulated when flr-4 is knocked down using RNAi. (C) The expression of GFP in cyp-35B1p::gfp was induced in the lower gut region when flr-4 is knocked down using RNAi. Head and tail are marked with H and T, respectively. Images are of worms 48 hours post L4. Images captured at 100x magnification. (D) The expression of ugt-16 and ugt-18 are not upregulated in sek-1(km4) to the same extent as in WT, when flr-4 is knocked down using RNAi. (E) The life span of flr-4(n2259) is suppressed to a greater extent (35% against 21%) compared to WT when these worms were grown on nhr-8 RNAi. (F) The expression of ugt-18, cyp-32B1 and gst-28 are not upregulated in nhr-8(ok186) to the same extent as in WT, when flr-4 is knocked down using RNAi. Error bar indicates SEM. ****P≤0.0001, ***P≤0.001, **P≤0.01, *P≤0.05, n.s. not significant, Student’s t test. Day 1 adult worms were used for RNA-seq and QRT-PCR. (G) The life span of WT worms does not change when vhp-1 is knocked down using RNAi. Life spans were performed at 20 oC. (PDF) [file pgen.1007608.s008.pdf]

A

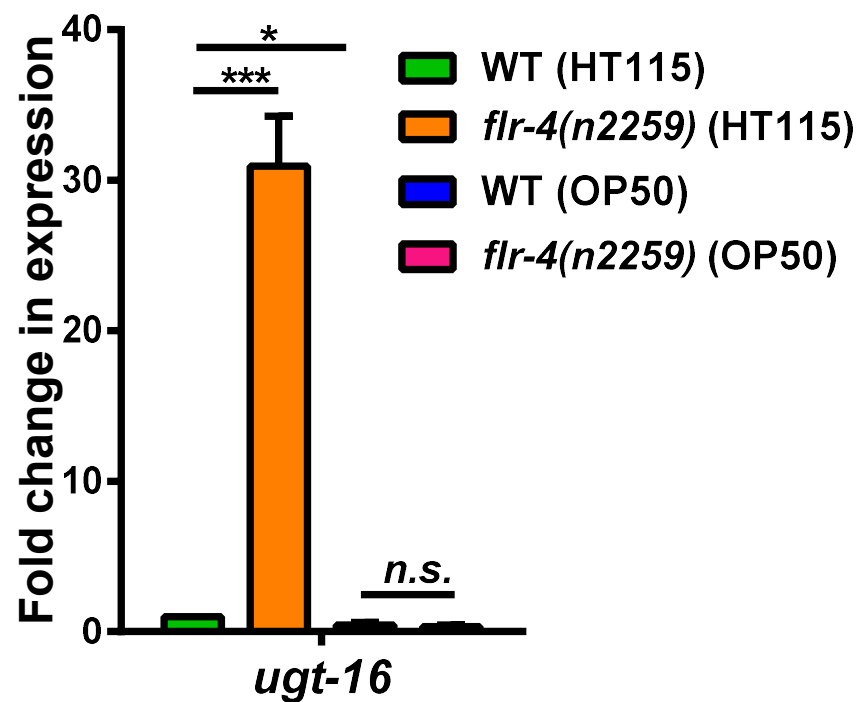

B

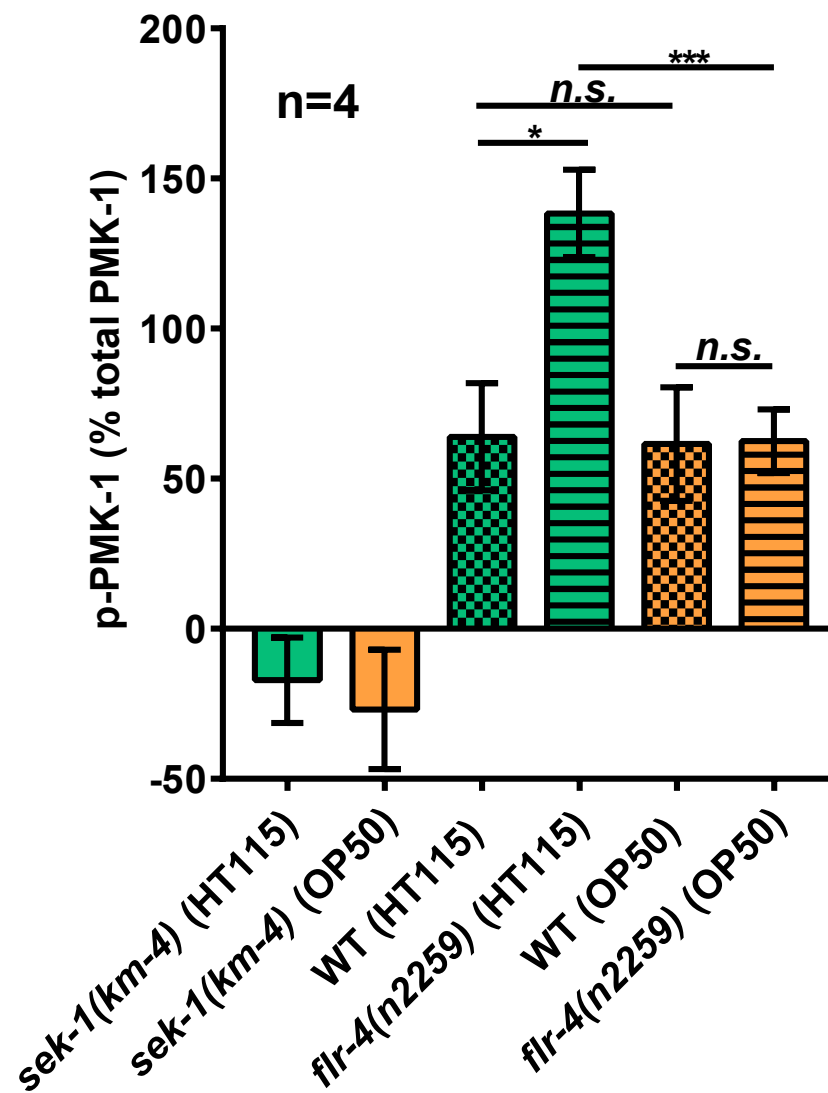

Supplement: S9 Fig — (A) QRT-PCR validation of RNA-seq data for ugt-16 which is upregulated only when flr-4(n2259) worms were fed HT115. (B) Quantitation of data of Fig 5D. The intensity of pPMK-1 and PMK-1 bands were normalized to beta-actin bands. Percent intensity of pPMK-1 with respect to (w.r.t.) total PMK is plotted. Average of four experiments shown. Error bars are SEM. ***P≤0.001, **P≤0.01, *P≤0.05, n.s. not significant, Student’s t test. Day 1 adult worms were used for QRT-PCR and western blot analysis. (PDF) [file pgen.1007608.s009.pdf]

Figure S10

**A**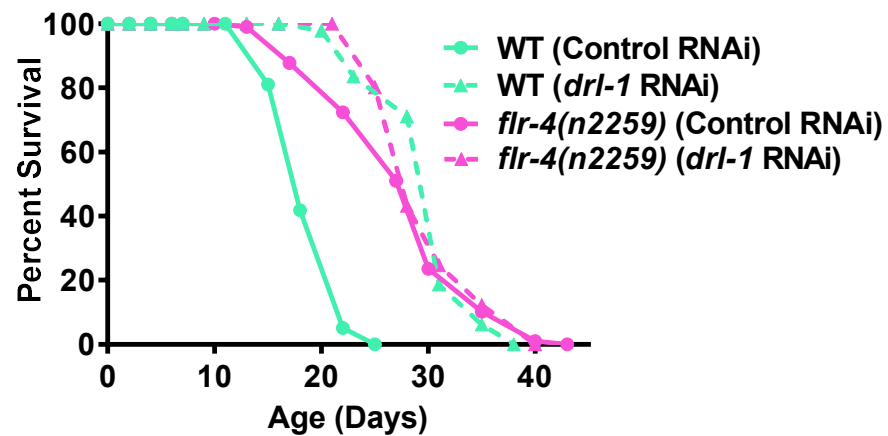**B**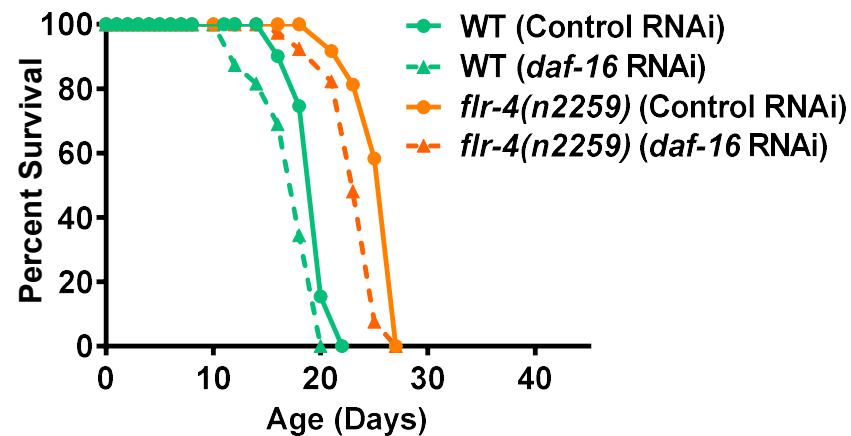**C**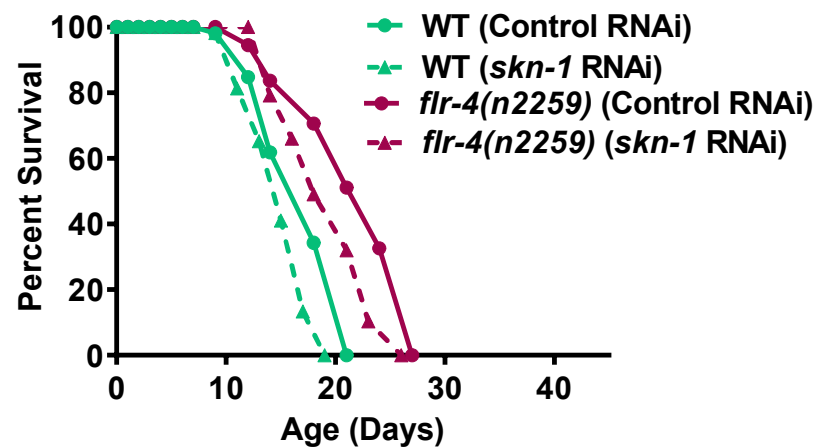**D**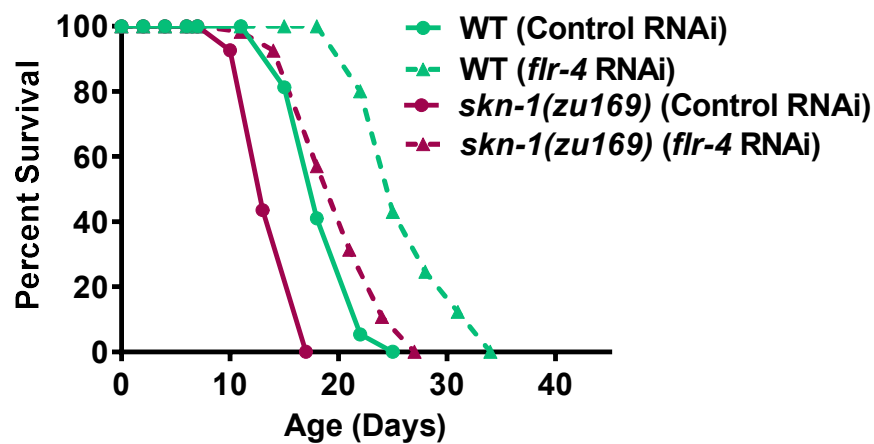

Supplement: S10 Fig — (A) Knocking down drl-1 by RNAi does not further prolong the extended life span of flr-4(n2259). (B) The daf-16 RNAi suppresses life span of WT and flr-4(n2259) to similar extent. (C) The skn-1 RNAi suppresses life span of WT and flr-4(n2259) to similar extent. (D) The flr-4 RNAi extends life span of WT and skn-1(zu169) to similar extent. Life spans were performed at 20 oC. (PDF) [file pgen.1007608.s010.pdf]

Figure S11

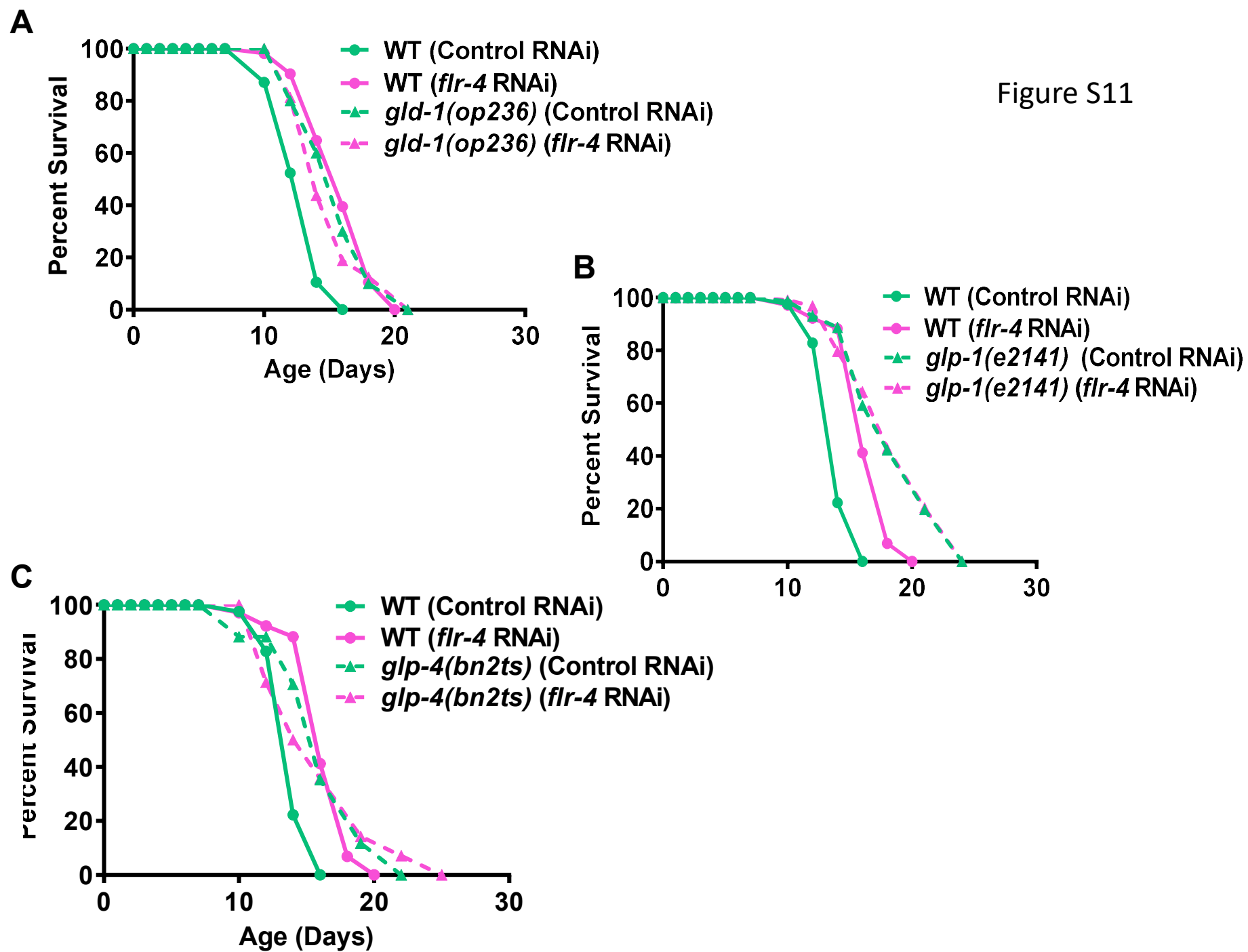

Supplement: S11 Fig — (A-C) Life span analysis was performed on different germline-defective mutants that were grown on control or flr-4 RNAi. The worms were maintained at 15 oC and life spans performed at 25 oC. (PDF) [file pgen.1007608.s011.pdf]

**A**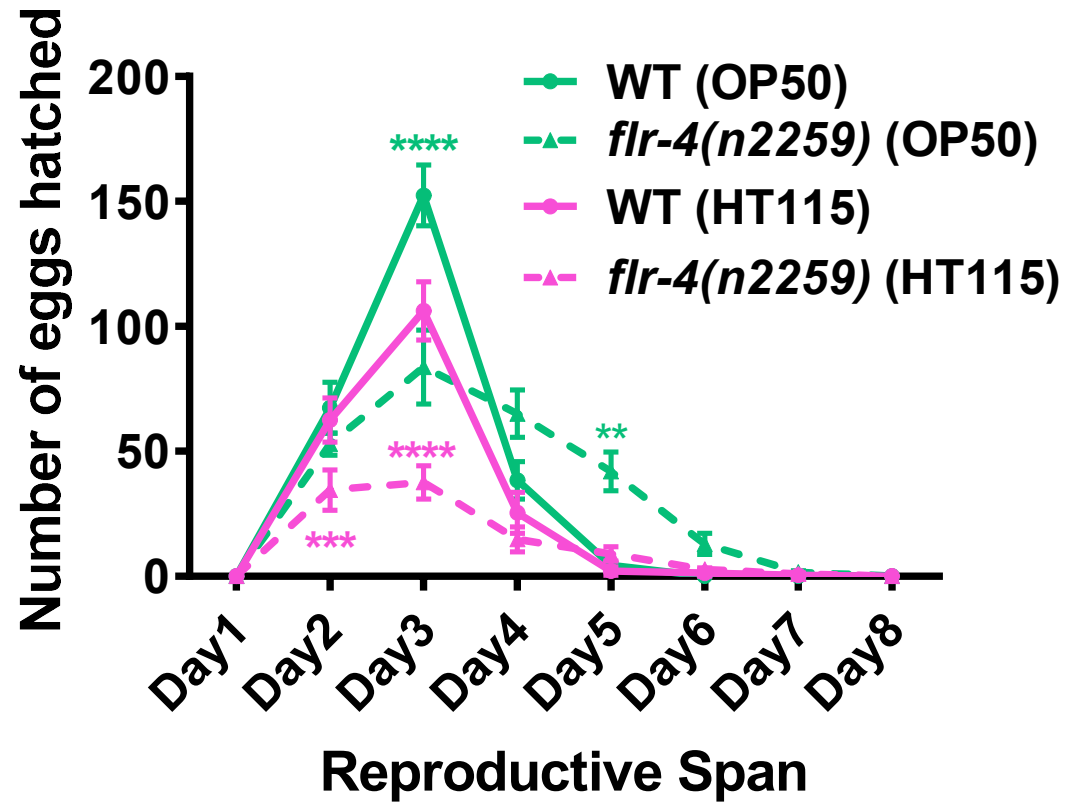**B**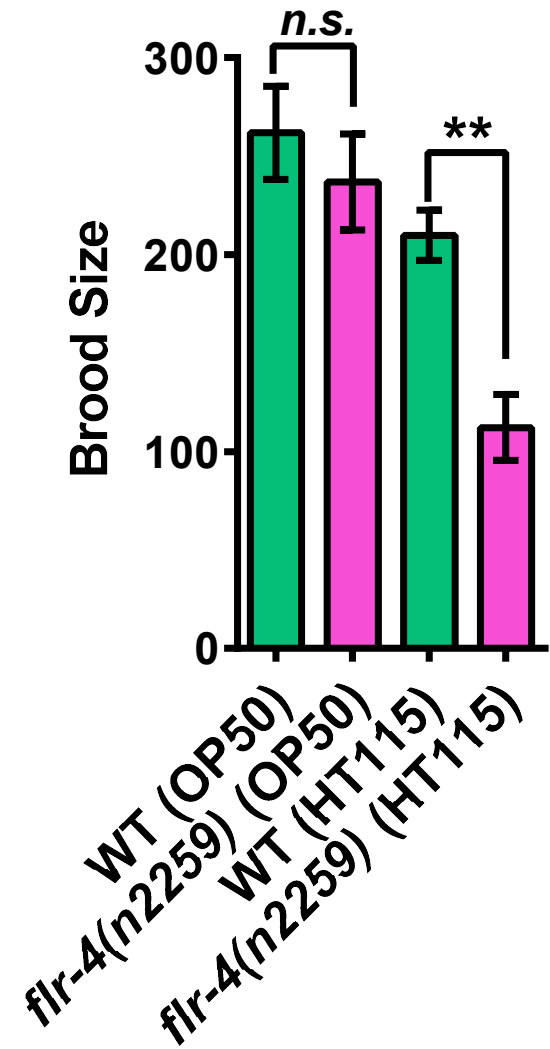

Figure S12

Supplement: S12 Fig — (A) Reproductive span analysis of WT or flr-4(n2259) grown on HT115 or OP50. Number of eggs that hatched are plotted against the number of days. (B) Total number of eggs hatched over the entire reproductive span in shown. Comparisons are made between the WT and mutant on a particular diet. Error bars are SEM. ***P≤0.001, **P≤0.01, *P≤0.05, n.s. not significant, Student’s t test. (PDF) [file pgen.1007608.s012.pdf]

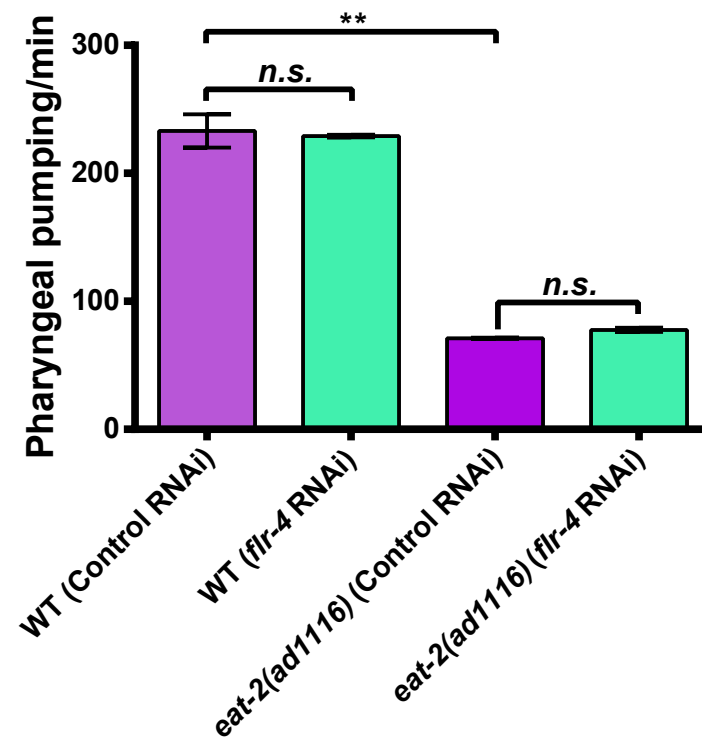

Supplement: S13 Fig — Error bars are SEM. **P≤0.01, n.s. not significant, Student’s t test. (PDF) [file pgen.1007608.s013.pdf]

Figure S14

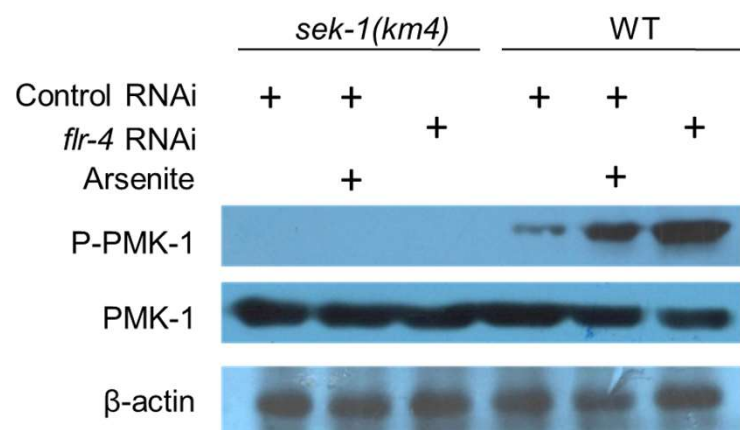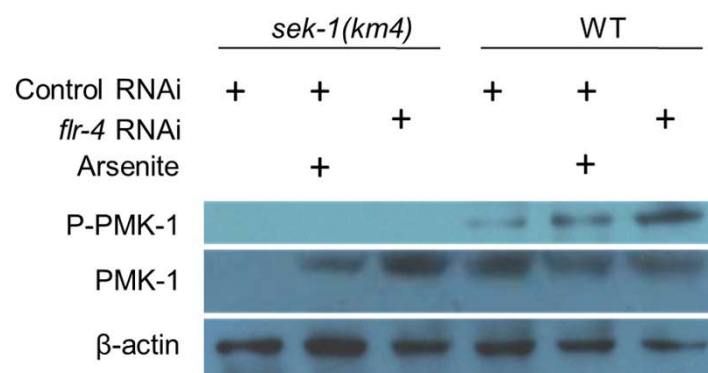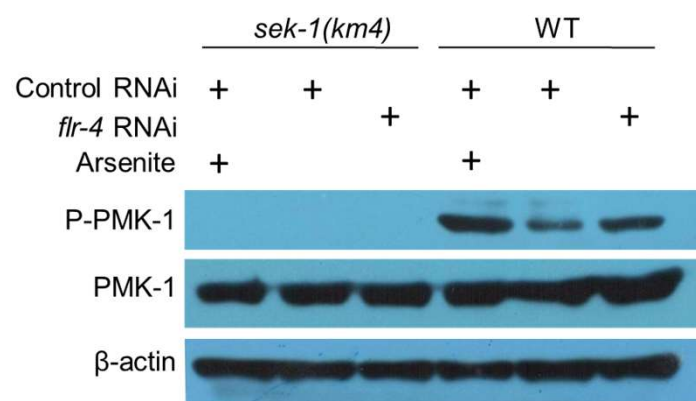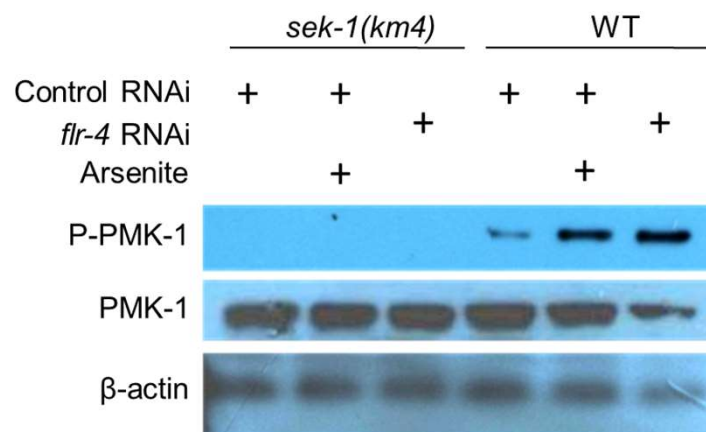

Supplement: S14 Fig — Refers to Fig 3B. (PDF) [file pgen.1007608.s014.pdf]

Figure S15

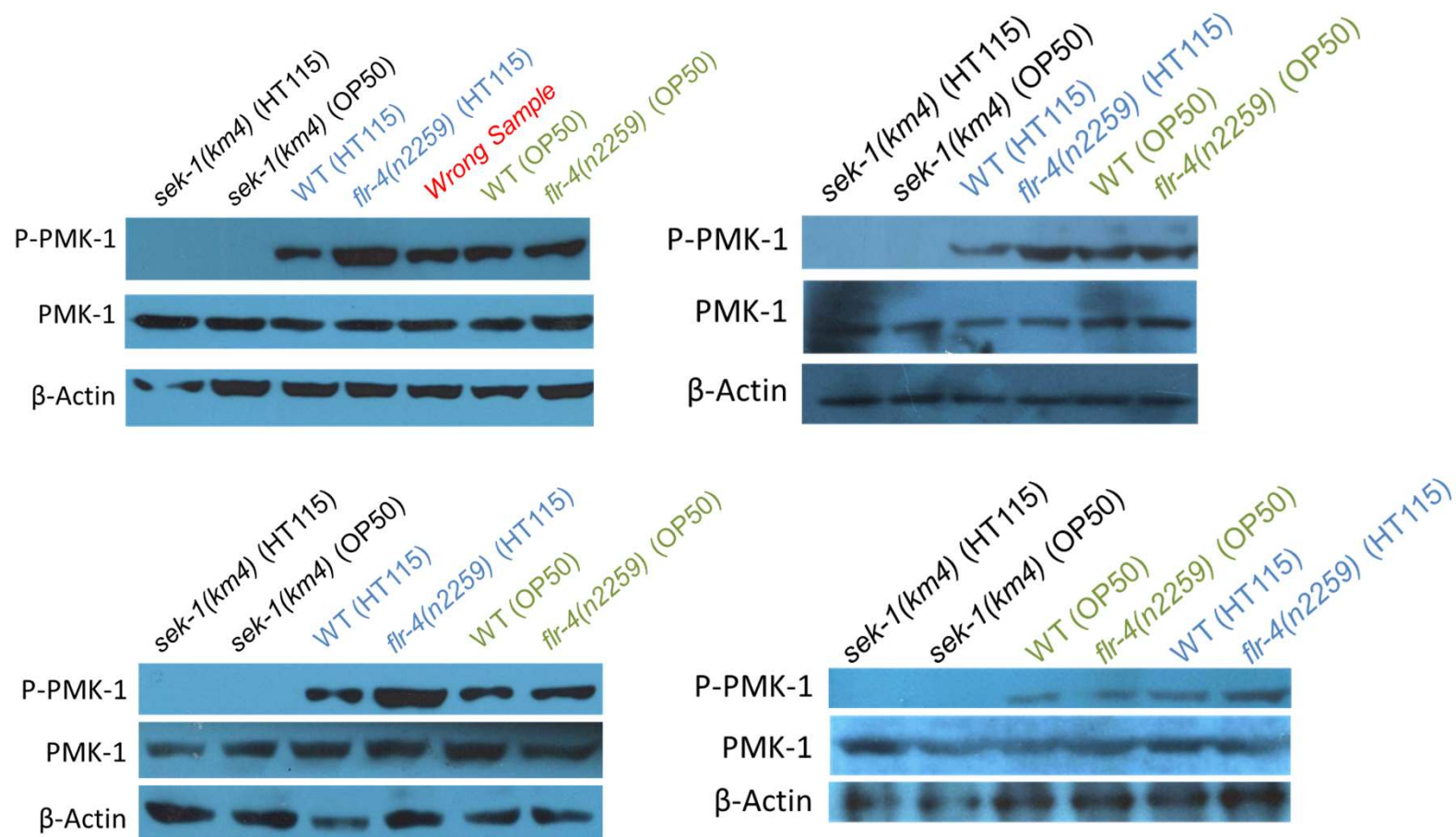

Supplement: S15 Fig — Refers to Figs 5D and S9B. (PDF) [file pgen.1007608.s015.pdf]

Figure S16

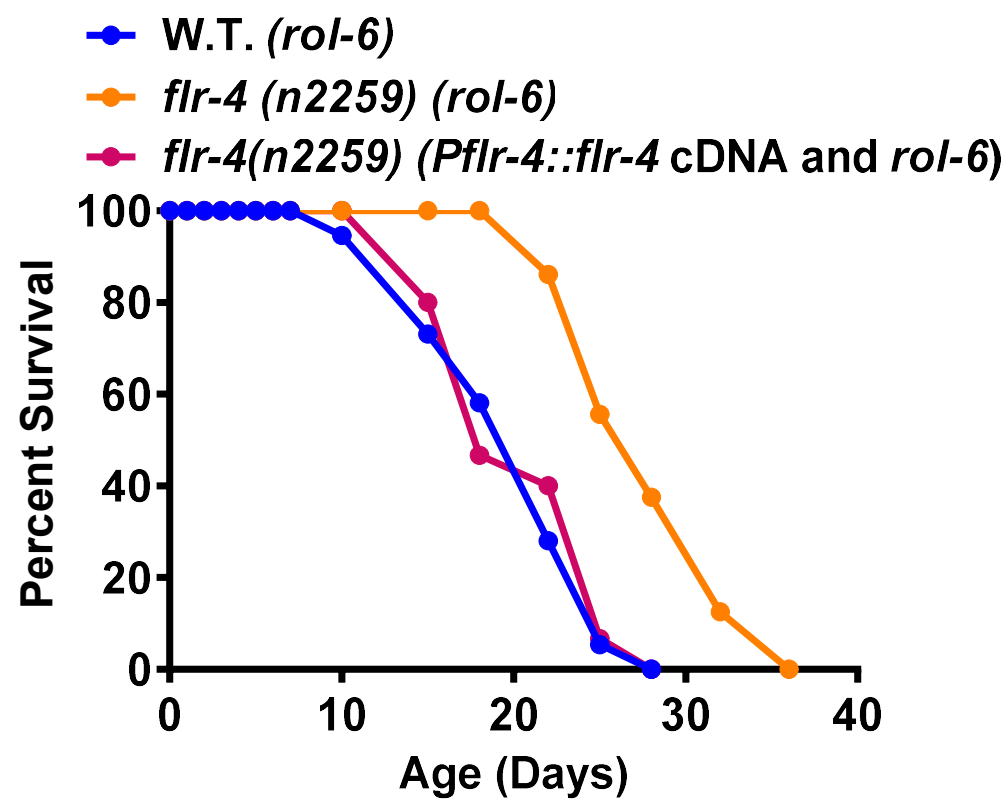

Supplement: S16 Fig — Transgenic worms were generated by injecting WT and flr-4(n2259) with pRF4 (rol-6) plasmid as well as flr-4(n2259) with Pflr-4::flr-4 cDNA along with pRF4. Life spans performed at 20 oC. (PDF) [file pgen.1007608.s016.pdf]
